# Cost and affordability of scaling up tuberculosis diagnosis using Xpert MTB/RIF testing in West Java, Indonesia

**DOI:** 10.1371/journal.pone.0264912

**Published:** 2022-03-10

**Authors:** Mardiati Nadjib, Retno Kusuma Dewi, Ery Setiawan, Tri Yunis Miko, Septiara Putri, Panji Fortuna Hadisoemarto, Euis Ratna Sari, Pujiyanto, Rani Martina, Lusi Nursilawati Syamsi

**Affiliations:** 1 Department of Health Policy and Administration, Faculty of Public Health Universitas Indonesia, Depok, Indonesia; 2 National TB Program, Ministry of Health Republic of Indonesia, Jakarta, Indonesia; 3 Center for Health Economics and Policy Studies, Universitas Indonesia, Depok, Indonesia; 4 Department of Epidemiology, Faculty of Public Health Universitas Indonesia, Depok, Indonesia; 5 Department of Public Health/Tuberculosis Working Group, Faculty of Medicine Universitas Padjadjaran, Bandung, Indonesia; 6 District Health Office, Depok Municipality, West Java, Indonesia; 7 Department of Internal Medicine, Sentra Medika Hospital, Depok, Indonesia; The University of Georgia, UNITED STATES

## Abstract

In Indonesia, a significant number of tuberculosis (TB) cases may be missed, due to the low sensitivity and specificity of the currently used diagnostic algorithm. In this regard, the rapid molecular test using Xpert MTB/RIF, which has recently been introduced in Indonesia, can improve case detection. Thus, this study determined the cost and affordability of incorporating Xpert MTB/RIF testing for TB diagnosis. For this purpose, we estimated the costs (from the health system and societal perspectives) of reaching the TB detection target in Depok municipality, and applied the findings to the West Java province of Indonesia. The resources available for the health and TB program were also analyzed to support the decision to scale up the TB diagnosis using Xpert MTB/RIF testing. According to the results, the unit cost for TB diagnosis per person was USD 27.22 and USD 70.16 from the health system and societal perspectives, respectively. To reach the target of 109,843 TB cases for the 2020–2024 time period, Depok municipality would need USD 2,989,927 and USD 2,549,455 from the health system viewpoint, assuming the machine’s lifespan of five and 10 years, respectively. Extrapolating these results to the West Java province, USD 56,353,833 would be necessary to test 2,076,413 cases from 2019 to 2024. However, in order to accelerate the case detection target up to 2024, West Java requires additional funds. The implication of the findings is that the central government must consider local capacity to accelerate TB case detection and ensure that the installation of Xpert MTB/RIF machines is included in the overall costs.

## Introduction

### Background

Although Indonesia has experienced noticeable progress in tuberculosis (TB) control over the last decade, TB remains one of the top four causes of death in the country [1]. For example, the estimated TB incidence among Indonesian adults (15 years old and above) in 2018 was 316 (95% CI: 288–345) per 100,000 population, which corresponds to a projected number of 845,000 (95% CI: 770,000–923,000) TB cases per year [2]. Additionally, only 570,289 cases were identified, whereas the rest was undetected or unreported [2].

TB incidence in Indonesia incurred a cost of USD 6.9 billion to the national economy, which may be owing to undetected and untreated cases [3]. Specifically, the two highest liabilities were derived from the loss of productivity due to premature death and illness, comprising USD 6.0 billion and USD 700 million, respectively [3]. Besides the need to address various factors associated with the TB problem, an urgent need has emerged for strategies that increase the detection rate for TB cases. In this regard, Indonesia aimed to reduce TB incidence to 190 per 100,000 adults by 2024, as explicitly articulated in its National Strategic Plan [4].

The World Health Organization (WHO) recommends the use of Xpert MTB/RIF (*Mycobacterium tuberculosis* [MTB] complex and resistance to rifampin [RIF]) testing instead of conventional microscopy and culture [5–7]. Given the wide coverage of health centers in Indonesia, optimizing the detection of TB cases using more sensitive and specific diagnostic tools is important. The National TB Program (NTP) in Indonesia integrates this recommendation for detecting TB cases in primary care. Through the Ministry of Health’s (MOH) Regulation No. 67/2016 regarding TB control, the Indonesian government stipulates that its laboratory network use Xpert MTB/RIF testing for the diagnosis of drug-sensitive TB, drug-resistant TB (DR-TB), and TB in people living with HIV/AIDS [8]. The currently used diagnostic algorithm is based on bacteriological examination by sputum smear microscopy (SSM) using the Ziehl–Neelsen direct light technique followed by clinical examination with or without chest radiography (CXR) [8].

Although molecular diagnostic tools for TB and DR-TB are available in 878 health facilities in 478 districts [4], the MOH has been planning to increase the number of Xpert MTB/RIF testing units in the country [9]. In this regard, various studies have addressed the issue of the cost-effectiveness of Xpert MTB/RIF testing as an important approach for decision-making in low- and middle-income countries [10–14]. However, little is known about whether this type of diagnostic test is affordable, especially at the sub-national level. Thus, a budget impact analysis is important because it can provide information on the financial consequences of scaling up the use of Xpert MTB/RIF testing in the country [15]. Based on this objective, this study assesses the cost and affordability of using Xpert MTB/RIF testing to diagnose TB in 27 districts of West Java province.

### Objective

The objective of this study is three-fold, namely, to (1) conduct a cost analysis of scaling up TB diagnosis using Xpert MTB/RIF testing; (2) estimate the budget impact for TB diagnosis during 2020–2024; and (3) determine the affordability of scaling up TB diagnosis using Xpert MTB/RIF testing in West Java province.

## Materials and methods

### Study setting

The study selected West Java (a high-burden province) as the study site, which had 156,143 incidences of TB in 2017 or 330 per 100,000 people (estimated case detection rate: 55%) [16]. This study was conducted in Bogor district, Cimahi municipality, and Depok municipality. The Bogor district had the highest number of notified TB patients among all of the districts in Indonesia, with 7,738 patients in 2014 and 10,405 cases in 2017, while Depok and Cimahi municipalities reported 3,734 and 1,802 TB cases, respectively [16].

### Costing study

In this study, the cost data was captured from the societal perspective. The health system costs were completed in 2018, whereas data for patient costs were collected in 2019. All of the costs were converted into USD in 2018 and 2019, at the rate of 13,850 rupiah and 14,000 rupiah, respectively. No discounting or inflation rate adjustment was applied. We also used the cost data to simulate the budget impact for West Java covering 27 districts, based on purchasing power parity (PPP) and the data published by the Central Bureau of Statistics [17].

Real world costing studies were carried out in 13 purposively selected health centers and one tertiary hospital across three districts (Bogor district, Depok municipality, and Cimahi municipality). Specifically, 10 health centers in Bogor district were selected to represent the provision of TB diagnosis in this high burden district using SSM and SSM plus CXR, while three health centers in neighboring high burden districts (Bogor district and Depok municipality) represented the provision of TB diagnosis using Xpert MTB/RIF testing [18]. We also selected the providers based on specific criteria: availability of qualified staff/physicians, accessibility, and the government priority area for West Java province. As for the data, it was gathered to represent the patient flow from the registration desk to the end point where the patients received their TB diagnosis. This estimation considered all possible modalities for TB diagnosis in the current algorithm [8]. Moreover, we included the hospital as part of our costing study to capture the costs of providing Xpert MTB/RIF testing for the cases in which there was no access/availability to such testing at the health center.

Additional data was collected from one health center and one hospital in Depok to obtain comprehensive direct medical costs, direct non-medical costs, and indirect medical costs, as suggested by the WHO’s guideline [19]. These facilities represent various characteristics in terms of costs (infrastructure) and demographic issues (urban/rural). We analyzed the costing data from both the district and province levels, and extrapolated such data to represent the budget consequence of providing Xpert MTB/RIF testing in Depok and West Java province.

Data on patient costs were collected using a questionnaire that measured direct medical costs, direct non-medical costs, and indirect costs (foregone earnings) [19]. Direct medical costs included costs for consultation, administration, laboratory testing, CXR examination, and expenses during inpatient care related to diagnostic testing at the hospital. Alternatively, non-medical costs comprised costs incurred on food, travel, and caregiver/guardian. Notably, the income losses of patients were calculated as part of indirect costs [19, 20].

The patient costs data were collected from one health center and one private hospital in Depok, the only facilities assigned to provide Xpert MTB/RIF testing in the city. We captured the patient’s experience in receiving the diagnostic TB tests, starting from their search for care based on initial symptoms to being referred to the final diagnosis using Xpert MTB/RIF testing. Specifically, we employed a non-probability sampling method by consecutively enrolling pulmonary TB cases from July to August 2019, and assumed that the data from 31 patients in the health center and 31 patients in the hospital would be sufficient for capturing their expenses.

The inclusion criterion comprised patients aged ≥15 years who were referred from other health care centers/clinics to the two designated facilities in order to conduct a TB diagnosis using Xpert MTB/RIF testing. Any patients from vulnerable groups (e.g., pregnant women, children, the elderly with comorbidities, etc.) or those who were unwilling to be interviewed were excluded from this study. Prior to the interviews, informed consent was obtained by each participant and his/her representative, while for the participants under the age of 18, parental consent was documented.

The health system costs were defined as the sum of all direct and indirect healthcare costs, including incidental one-off costs (e.g., purchasing an Xpert MTB/RIF machine) as well as maintenance and routine costs (e.g., operational costs, consumables, staff costs, and other operational costs). The consumables cost was considered as a variable cost measured per each diagnosis procedure. We also estimated the annual value of equipment cost and then divided it by the total annual utilization for certain activities such as total SSM, CXR, and Xpert MTB/RIF testing. Over a 3-year period, the costs for the Xpert MTB/RIF machine, cartridge, and maintenance were USD 17,000, 10, and 7,902, respectively, per machine [21]. In addition, we estimated the number of annual tests per day based on the MOH’s suggestion regarding the capacity and utilization of the machine. In this regard, less than 10 tests per day were considered as low, 10–50 tests per day were considered as moderate, and more than 50 tests per day were considered as high. We assumed that the Xpert MTB/RIF machine would perform three modules per day or 12 expected tests per day. Based on the findings, in Depok, the number of Xpert MTB/RIF tests was 1,925 per year or approximately eight tests per day. For the analysis, we assumed that the health centers could perform roughly 2880–3000 tests annually, according to 20–21 working days per month.

Finally, the unit of analysis was presented as per patient value. Hence, the unit cost represented the amount of money spent by each household for one diagnostic procedure, including both health system and patient costs. Moreover, the cost per case was derived from the cost analysis and the number of cases detected for each of the current modalities, while the total annual cost of using Xpert MTB/RIF testing was calculated by multiplying the unit cost by the number of tests required per year for the target population. We also calculated the costs for SSM to determine the financial consequences of scaling up the TB diagnosis using Xpert MTB/RIF testing.

### Number of TB diagnostic tests needed and affordability

The data from the Depok municipality regarding the number of TB presumptive cases as well as the number of SSM and Xpert MTB/RIF tests for the 2016–2018 time period were extracted from the national electronic TB register. The number of Xpert MTB/RIF tests in the study area partly contributed by transporting the specimens from other health centers. Since this approach has not been launched as a national program, for this study, we focused on capturing on-site Xpert MTB/RIF testing (out-patient visits) and expecting more health centers to provide such testing in West Java.

A budget impact analysis for the 2019–2024 time period was conducted based on the cost data collected and the number of cases detected by the West Java Provincial Health Office (PHO). Conceptually, affordability was assessed by comparing the costs of Xpert MTB/RIF testing relative to currently available funds [6]. To determine the affordability of the proposed budget, this study compared the cost of the targeted Xpert MTB/RIF testing relative to total health expenditures (specifically on TB care and control). Information was derived from the District Health Office (DHO) and the Local Government Medium-Term Development Plan for 2018–2023 [22]. In summary, the data sources comprised primary data and official documents (Table 1).

**Table 1:** Methods used to estimate the number of TB tests and costs in Depok municipality and West Java province.

Variable	Description	Source
**Presumptive TB cases**	All people with TB signs and symptoms	DHO Depok and PHO West Java
• **SSM**	Suspected TB with SSM test if negative, followed by a chest x-ray (CXR)	DHO Depok and PHO West Java
• **Xpert MTB/RIF**	Presumptive TB using Xpert MTB/RIF testing	DHO Depok and PHO West Java
**Estimated number of TB cases**	Estimated number of cases in 2016–2019 (existing) and 2020–2024	DHO Depok and PHO West Java
**Case projection based on the scenario**		
• **Case with symptoms and SSM test**	Coverage of TB cases with SSM tests	DHO Depok and PHO West Java
• **Case with symptoms and Xpert MTB/RIF testing**	Coverage of TB cases with Xpert MTB/RIF testing	DHO Depok and PHO West Java
**Cost of diagnosis**	Cost of TB diagnosis in the health center or hospital for SSM and Xpert MTB/RIF testing	Primary data
• **Health system perspective**	Cost of healthcare component related to TB diagnosis	Primary data
• **Patient perspective**	Patient costs, including transportation and other direct non-medical costs	Primary data
• **Societal perspective**	Direct medical, direct non-medical, and indirect costs for TB diagnostic tests	Primary data
**Budget impact**	Resource needed for Xpert MTB/RIF testing for the period 2020–2024	Estimate for the period 2020–2024 is based on costs (health system and patient costs) and TB case projection for West Java
**Financing (affordability)**	Resource available for the TB program/affordability	
• **Public funding**	Government financial support for the TB program and the health sector at the sub-national level	District Health Account Depok; West Java Province Medium-term Development Plan; and public funding documents

### Budget impact scenario and uncertainty

We estimated the cost of providing Xpert MTB/RIF testing in Depok and West Java by multiplying the unit cost and number of targeted cases. Based on the findings, the proportions of the cases using SSM and Xpert MTB/RIF testing included: 60% by SSM and 40% by Xpert MTB/RIF (60:40) in 2019, 50:50 in 2020, 48:52 in 2021, 45:55 in 2022, 42:58 in 2023, and 40:60 in 2024 [23]. In addition, a budget impact scenario was undertaken deterministically regarding the expected years of useful life for a Xpert MTB/RIF machine at 10 years, instead of five years. We also assessed the uncertainty of certain parameters incurred in cost calculations, including: costs, number of TB cases, and total costs (scenario).

### Ethics

This study was approved by the Research and Community Engagement Ethical Committee in the Faculty of Public Health at Universitas Indonesia (Approval No. 555/UN2.F10/PPM.00.02/2019).

## Results

### Unit cost of TB diagnostic tests in Depok

The average costs of the laboratory tests provided by the health center and hospital were USD 2.02 per test for the SSM, USD 8.86 for the CXR, and USD 27.22 for the Xpert MTB/RIF, including maintenance costs (see Table 2). The costs also included the annual cost of the staff performing such tests. Based on the findings, the Xpert MTB/RIF machine, cartridge, and maintenance costs were the three largest cost components for Xpert MTB/RIF testing, which are currently subsidized by the central government.

**Table 2:** Average cost per person tested (USD).

Cost perspective	Polyclinic	CXR	SSM	Xpert MTB/RIF
**Health system**	1.90	8.86	2.02	27.22
**Patient (95% CI)**				
**Direct non-medical**	-	6.04	0.93	11.55
		(3.73–8.36)		(8.38–14.71)
**Indirect**	-	16.14	2.86	31.39
		(7.45–24.83)		(24.49–38.29)
**Total**	-	22.19	3.79	42.94
				(33.97–51.90)
		(12.10–32.26)		
**Societal (95% CI)**				
**Lab only + direct non-medical**	1.90	14.90	2.95	38.77
		(13.30–16.50)		(34.35–40.68)
**Societal (95% CI)**	1.90	31.04	5.81	70.16
**Lab only + direct non-medical + indirect**				
				(59.94–77.87)
		(24.07–38.02)		

The average patient cost (the health center and hospital) was USD 3.79 for the SSM, USD 22.19 for the CXR, and USD 42.94 for the Xpert MTB/RIF. As for the indirect costs (foregone earnings), they accounted for 32% to 58% of the total patient costs. It should be noted that the majority of the patients were covered by social insurance schemes. Thus, the direct medical costs paid out-of-pocket (in most cases) were relatively low. However, such costs were higher for those who were treated at the hospital and required inpatient care. Regarding the direct non-medical costs for transportation, meals, and caregiver/guardian, they were relatively lower than the other components, although the costs (especially transportation) were considered as the main challenge for accessing care among the patients from both rural and urban areas [20]. Moreover, a statistical analysis revealed that the type of facility and transportation was associated with a higher average cost (see Table 3).

**Table 3:** Cost variations by patients’ socio-demographic variables.

Category		N (%)	Mean in USD (SD)	p-value
**Age** ***				0.0517
	≤20	4 (6%)	42.20 (6.35)	
	21–40	31 (50%)	41.36 (13.08)	
	41–60	19 (31%)	52.16 (16.60)	
	≥61	8 (13%)	41.15 (12.18)	
**Type of health facility** *				
	PHC	31 (50%)	39.69 (12.20)	0.0020
	Hospital	31 (50%)	49.45 (15.16)	
**Health status (BMI)** *				
	Min-max	13.84–29.78	0.0439	
	Median	18.34		
	Average (SD)	19.11 (3.3)		
**Referred/not** **				0.1794
	Enrolled as a new patient	33 (53%)	43.53 (15.46)	
	Referred from another HC	28 (45%)	46.37 (13.36)	
**Transportation mode** ****				0.0275
	Public transportation	16 (25.8%)	45.96 (15.43)	
	Private car	7 (11.29%)	59.11 (13.94)	
	Motorcycle	38 (61.29)	41.49 (12.93)	

Notes

* Mann Whitney

** Spearman

*** One-way ANOVA

**** Kruskal–Wallis; ^a^ SD = standard deviation.

The number of positive bacteriological cases using SSM in 2016 was 1,372, while using CXR on negative bacteriological cases with positive clinical symptoms resulted in an additional 1,451 clinical TB cases. From the health system perspective, the costs incurred by Depok in 2016 were USD 15,528 for SSM and USD 83,635 for SSM-CXR. From the societal perspective, the costs were USD 22,677 and USD 151,818, respectively. The largest cost components for the SSM, CXR, and Xpert MTB/RIF were direct medical costs, each of which contributed 69%, 59%, and 68% of the total costs, respectively. This was followed by transportation costs that (on average) contributed 30% of the total costs. The findings for the Xpert MTB/RIF testing gave a 26.7% positivity rate, with 870 resistant cases in 2018, out of the 3,400 cases. The number of resistant cases that were later referred to hospitals were 32 in 2017 and 65 in 2018. Regarding the costs from the health system and societal perspectives, and the cost per case detected using Xpert MTB/RIF testing in the 2016–2018 time period, see Table 4.

**Table 4:** Number of TB cases, number of tests, and costs in Depok, 2016–2018.

	2016	2017	2018
**Total cases tested**	7,687	6,969	8,205
• **Xpert MTB/RIF**	0	742	3,400
• **SSM**	7,687	6,227	4,805
Number of cases			
**Total TB cases**	2,823	3,734	3,799
**Total pulmonary TB**	NA	3,106	3,246
**TB new cases with AFB (+)**	1,372	1,580	1,483
* SSM	1,372	1,346	613
* Xpert MTB/RIF	NA	234	870
**TB new cases with clinical symptoms**	1,451	1,260	1,607
**TB-resistant**	NA	32	65
Total health system cost **(USD)**			
**SSM**	15,528	12,579	9,706
**Xpert MTB/RIF**		21,132	92,548
**SSM-CXR**	83,635	67,750	52,278
Total societal **cost (USD)**			
**SSM**	22,677	18,370	14,175
**Xpert MTB/RIF**		28,767	131,818
**SSM-CXR**	151,818	122,983	94,899
Cost per case detected **(USD)**			
**SSM**	11.32	9.35	15.83
**Xpert MTB/RIF**		90.31	111.30
**SSM-CXR**	57.64	53.77	32.53

Note: NA = not available.

### Budget impact for TB tests in Depok, 2019–2024

The case detection target for Depok was established by the PHO, increasing from 6,268 in 2019 to 6,601 in 2024 (see Table 5). Thus, we applied this increase in Xpert MTB/RIF testing from 2019 to 2024. This table also shows the number of cases using such testing and its costs. Specifically, in 2019, the total cost was USD 409,554 and USD 584,816, from the health system and societal perspectives, respectively. However, it is expected to increase to USD 610,955 for the health system perspective and USD 871,235 for the societal perspective by 2024. The historical data from the study area also showed a positivity rate of 26.7% for Xpert MTB/RIF testing and 10% for SSM, effectively yielding positivity values of 3,615 in 2019 and 5,710 in 2024.

**Table 5:** Estimated costs for the TB diagnostic tests in Depok, 2019–2024.

	Type of Testing	2019	2020	2021	2022	2023	2024
	Type of test (SSM: Xpert MTB/RIF)	60:40	50:50	48:52	45:55	42:58	40:60
**Number of cases**	TB presumptive	33,847	33,890	33,890	35,726	35,689	35,645
	TB cases estimated	6,965	6,973	6,973	6,965	6,956	6,948
	Case detection target	6,268	6,276	6,276	6,616	6,609	6,601
**Estimated number of cases tested**	SSM	20,308	16,945	16,267	16,077	14,989	14,258
	Xpert MTB/RIF	13,539	16,945	17,623	19,649	20,700	21,387
**Health system perspective cost (USD)** ^*^	SSM	41,022	34,229	32,859	32,476	30,278	28,801
	Xpert MTB/RIF	368,532	461,243	479,698	534,846	563,454	582,154
	**Total**	**409,554**	**495,472**	**512,557**	**567,321**	**593,732**	**610,955**
**Health system perspective cost (USD)** ^**^	SSM	41,022	34,229	32,859	32,476	30,278	28,801
	Xpert MTB/RIF	314,240	393,293	409,030	456,053	480,447	496,392
	**Total**	**355,262**	**427,522**	**441,889**	**488,529**	**510,725**	**525,193**
**Societal perspective cost (USD)** ^*^^*^	SSM	59,909	49,988	47,988	47,427	44,218	42,061
	Xpert MTB/RIF	524,907	656,958	683,244	761,792	802,539	829,174
	**Total**	**584,816**	**706,945**	**731,231**	**809,219**	**846,757**	**871,235**
**Societal perspective cost (USD)** ^**^	SSM	59,909	49,988	47,988	47,427	44,218	42,061
	Xpert MTB/RIF	470,616	589,008	612,575	682,999	719,532	743,412
	**Total**	**530,524**	**638,996**	**660,563**	**730,426**	**763,750**	**785,473**
**Number of positive cases**	SSM	2,031	1,695	1,627	1,608	1,499	1,426
	Xpert MTB/RIF	3,615	4,524	4,705	5,246	5,527	5,710
	**Total**	**5,646**	**6,219**	**6,332**	**6,854**	**7,026**	**7,136**

Notes

* Useful life of Xpert MTB/RIF machine = 5 years

** Useful life of Xpert MTB/RIF machine = 10 years.

During this study, there were only two machines. However, in order to achieve the target set up by the PHO, six devices were required. Additionally, to reach the target of 109,843 TB cases detected using Xpert MTB/RIF testing in the 2019–2024 time period, Depok would need USD 2,989,927 and USD 2,549,455, assuming the useful life of the machine is five years and 10 years, respectively. According to the National Strategic Plan, by 2024, 75% of the tests will use Xpert MTB/RIF testing. Hence, for 2024 only, Depok would require USD 727,701 and USD 620,497, with the useful life of the machine at five years and 10 years, respectively [4].

### Affordability in Depok

Based on the findings, spending on TB programs in Depok generally increased, with the majority of the funds dedicated to monitoring drug compliance and supporting outreach programs. The TB tests, especially those using Xpert MTB/RIF testing, were highly subsidized by the central government, including support from the Global Fund [24]. However, the Global Fund support will end in 2023, after which it will become a challenge to continue these efforts without the government replacing such support (as an exit strategy), even for a region with high fiscal capacity such as Depok.

Table 6 shows that out of approximately USD 30 million in support for the health sector in Depok per year, spending on the TB program was relatively low, i.e., USD 77,326 and USD 128,929 in 2017 and 2018, respectively. This is an interesting finding, considering that TB is a priority public health issue in Depok.

**Table 6:** Funding for the TB program in Depok, 2017–2018.

Component of Funding	2017		2018	
	USD	%	USD	%
**Funding for TB by component**				
**Community involvement/education**	4,953	6.40	3,416	2.64
**Monitoring TB drug compliance**	50,763	65.65	62,447	48.44
**Outreach program**	8,220	10.63	10,816	8.39
**Cartridge** *	13,390	17.32	52,250	40.53
**Total**	**77,326**	**100**	**128,929**	**100**
**Funding for all health programs by source**			
**District budget** **	23,175,801	81.20	25,276,235	79.41
**Province budget**	814,708	2.85	1,211,896	3.81
Central budget ***	1,366,992	4.79	1,435,720	4.51
**Donor support**	13,390	0.05	522,157	1.64
**Subsidies for the poor**	3,171,801	11.11	3,385,154	10.63
**Total**	**28,542,692**	**100**	**31,831,162**	**100**

Note

* Central level contribution

** Including transfer funds from the central level; ** In kind and direct subsidy.

### Budget impact analysis of the TB diagnosis program in West Java

West Java includes 27 districts/cities with high to very high fiscal capacities, and two with low regional fiscal capacities [25]. Hence, a budget impact analysis for West Java for the next five years (2020–2024) was developed, based on our cost data (with an adjustment through PPP) and the PHO targets for the estimated number of TB cases.

Assuming that Xpert MTB/RIF testing with four modules was used, the optimal capacity would be 2,880–3,000 tests per year (with routine calibration), according to 20–21 working days per month. Hence, in order to achieve 50% testing of the aggregate presumptive cases, a total of 108 machines in West Java was required in 2019. However, if the number of Xpert MTB/RIF tests was increased by 10% in 2024, 136 machines would be required. To detect all targeted cases in West Java using Xpert MTB/RIF testing for the 2019–2024 time period, as much as USD 56,353,833 will be required (see Table 7). Based on the National Strategic Plan, which stated that 75% of the tests will use Xpert MTB/RIF testing in 2024, West Java province should allocate an additional USD 1.8 million that year.

**Table 7:** Costs needed in West Java, 2020–2024.

Category		Year				
	2019	2020	2021	2022	2023	2024
**Very high fiscal index (n = 12)**						
Presumptive TB cases	422,275	422,777	422,777	445,738	445,230	444,685
Estimated TB cases	86,890	86,992	86,992	86,890	86,785	86,682
Case finding target	78,199	78,292	78,292	82,544	82,450	82,349
Number of cases using SSM	211,137	211,388	202,933	200,582	186,997	177,874
Number of cases using Xpert MTB/RIF testing	211,137	211,388	219,844	245,156	258,233	266,811
Total costs for SSM (USD)	615,103	476,300	457,248	451,949	421,341	400,787
Total costs for Xpert MTB/RIF testing (USD)	8,084,575	6,260,217	6,510,626	7,260,198	7,647,538	7,901,573
Average cost for Xpert MTB/RIF testing per district (USD)	673,715	521,685	542,552	605,016	637,295	658,464
**High fiscal index (n = 9)**						
Presumptive TB cases	152,010	152,194	152,194	155,050	160,261	160,072
Estimated TB cases	31,277	31,314	31,314	31,277	31,240	31,205
Case finding target	28,150	28,184	28,184	28,713	29,678	29,643
Number of cases using SSM	76,005	76,097	73,053	69,773	67,310	64,029
Number of cases using Xpert MTB/RIF testing	76,005	76,097	79,141	85,278	92,951	96,043
Total costs for SSM (USD)	134,585	134,747	129,357	124,266	119,187	113,377
Total costs for Xpert MTB/RIF testing (USD)	1,768,904	1,771,040	1,841,882	1,996,233	2,163,299	2,235,245
Average cost for Xpert MTB/RIF testing per district (USD)	196,545	196,782	204,654	221,804	240,367	248,361
**Moderate fiscal index (n = 4)**						
Presumptive TB cases	40,198	40,246	40,246	42,439	42,390	42,331
Estimated TB cases	8,272	8,282	8,282	8,272	8,262	8,253
Case finding target	6,488	7,453	7,453	7,859	7,850	7,839
Number of cases using SSM	20,099	20,123	19,318	19,097	17,804	16,932
Number of cases using Xpert MTB/RIF testing	20,099	20,123	20,928	23,341	24,586	25,398
Total costs for SSM (USD)	39,158	39,205	37,637	37,207	34,687	32,988
Total costs for Xpert MTB/RIF testing (USD)	514,675	515,293	535,904	597,707	629,593	650,373
Average cost for Xpert MTB/RIF testing per district (USD)	128,669	128,823	133,976	149,427	157,398	162,593
**Low fiscal index (n = 2)**						
Presumptive TB cases	7,128	7,139	7,139	7,528	7,517	7,511
Estimated TB cases	1,467	1,469	1,469	1,467	1,465	1,464
Case finding target	1,320	1,322	1,322	1,394	1,392	1,391
Number of cases using SSM	3,564	3,569	3,427	3,387	3,157	3,005
Number of cases using Xpert MTB/RIF testing	3,564	3,569	3,712	4,140	4,360	4,507
Total costs for SSM (USD)	6,708	6,718	6,450	6,376	5,942	5,655
Total costs for Xpert MTB/RIF testing (USD)	88,168	88,304	91,836	102,420	107,849	111,492
Average cost for Xpert MTB/RIF testing per district (USD)	44,084	44,152	45,918	51,210	53,924	55,746
**West Java (n = 27)**						
Presumptive TB cases	621,610	622,355	622,355	650,754	655,398	654,599
Estimated TB cases	127,906	128,057	128,057	127,906	127,752	127,604
Case finding target	115,113	115,251	115,251	120,510	121,370	121,222
Number of cases using SSM	310,805	311,178	298,731	292,839	275,267	261,840
Number of cases using Xpert MTB/RIF testing	310,805	311,178	323,625	357,915	380,131	392,759
Total costs for SSM (USD)	652,691	653,473	627,334	614,963	578,061	549,863
Total costs for Xpert MTB/RIF testing (USD)	8,435,250	8,445,363	8,783,177	9,713,805	10,316,751	10,659,487
Average cost for Xpert MTB/RIF testing per district (USD)	312,417	312,791	325,303	359,771	382,102	394,796

Finally, in terms of uncertainty, we assessed the range values around the mean using standard deviation, as presented in Supplementary Material 1. We calculated such values from the variations in the cities/districts (n = 27) in West Java. According to the findings, there were substantial variations between these areas that affected the mean values for the costs adjusted, suspected cases, and the total costs for TCM and SSM. Moreover, we presented the total costs (categorized by fiscal index) to specify the differences in the included parameters.

### Affordability at the province level

By tracking down the data, we found that the total health budget in West Java was USD 174,250,595 in 2017, while the budget for the TB program (excluding Xpert MTB/RIF testing) was USD 7,449,826 (see Table 8), of which 51.9% was supported by donors (mainly the Global Fund) [26, 27]. This indicated that the TB program activities were mainly relying on donor support.

**Table 8:** Funding for TB in West Java, 2017.

Source	USD	%
**Central government** *	3,257,032	43.72
**Provincial government**	5,676	0.07
**District government**	317,227	4.26
**Donor**	3,869,891	51.95
**Total**	7,449,826	100
**Funding for health in total in 2017 (USD)** **	**174,250,595**	
**Funding for TB (in %) to total health budget**	**4%**	

Notes

* Including fund channeling central level support to districts, excluding capital

** All government-level sources.

Finally, the budget requisition for Xpert MTB/RIF testing in West Java in 2019 was USD 8,435,250 and it will increase to USD 10,316,751 in 2023, far exceeding its budget for the communicable disease programs (USD 606,200 in 2019 and USD 1,132,139 in 2023) [22]. However, with support from the MOH on laboratory infrastructure, and the budget for human resources and operational costs remaining under local government control, it seems unrealistic based on the evidence that only 4% of the health funds in West Java is dedicated toward the TB program (see Table 8).

## Discussion

Our study demonstrated that the cost of expanding the use of Xpert MTB/RIF testing for TB diagnosis is substantially larger, compared to the use of SSM alone or in combination with CXR. Interestingly, Xpert MTB/RIF testing not only imposed significant costs from the health system perspective, but also from the patients’ viewpoints, mainly because of the high cost of the machines, the patients’ costs (e.g., transportation and productivity loss) incurred, and the fact that only two sites provided this rapid test. Conforming to other studies, our findings showed that the rate of laboratory-confirmed TB diagnosis was considerably low, due to the poor quality of sputum, the troublesome path of diagnosis, and inaccurate reporting [28]. Meanwhile, SSM (the recommended test according to the Indonesian TB control guidelines) has a relatively lower sensitivity, while the gold standard mycobacterial culture test requires laboratory infrastructures that are not widely available in the country, with test results that return within four weeks. Conversely, rapid molecular tests, such as Xpert MTB/RIF testing, have a higher sensitivity (56%–74% vs. 88%) and specificity (91%–92% vs. 99%) [29, 30] than SSM, with a turnaround time as short as one day [31]. Hence, the use of Xpert MTB/RIF testing will increase diagnostic accuracy and eliminate any delays associated with the use of culture.

Furthermore, the use of Xpert MTB/RIF testing can give an additional yield of 16% for TB cases and 2% for cases of rifampicin-resistant TB, compared to using SSM alone. Assuming that each TB patient can infect up to 10 people within 18 months [30], using Xpert MTB/RIF testing can prevent as many as 16% of the new diagnosed cases. Consequently, the use of Xpert MTB/RIF testing could save economic losses [30]. Such testing can also reduce treatment costs, given the high cost of multi-drug-resistant tuberculosis or MDR-TB treatment [32]. However, testing all presumptive TB patients will significantly increase costs and be challenging, as asserted by the WHO [33]. Our study demonstrated that for West Java (a province with high TB prevalence), the cost of expanding the use of Xpert MTB/RIF testing would amount to USD 10,659,487 in 2024. Based on this statistic, Indonesia would face even higher budget requisitions to cover the targets for all 34 provinces.

In an effort to eliminate TB in Indonesia, which is predicted to be achieved by 2035, accelerated case detection with the help of Xpert MTB/RIF testing is expected to provide treatment for TB, MDR-TB, and TB-HIV cases. However, it needs operational improvement, including the recording and reporting of cases. In a related study of 44 health facilities in Indonesia, the examination of TB diagnosis using Xpert MTB/RIF testing produced a finding of 3.2% for DR-TB cases, which were followed up for further examination and treatment of MDR-TB [29]. Our study also revealed an increased number of cases following the introduction of Xpert MTB/RIF testing in 2017, after which the patients were referred to hospitals that provided MDR-TB treatment.

Under Indonesia’s decentralized system, the local government mostly finances the programmatic operational costs, while the provisions of supplies and infrastructure development are usually supported by the central government. In 2018, only 40% of the USD 294 million needed to finance the TB program in Indonesia was available, with the Global Fund providing close to 55% of the USD 35 million in international funding committed to Indonesia [34]. Considering the significant amount of support from the Global Fund and the heavy reliance on the central government for TB program financing, shifting the burden of financing Xpert MTB/RIF testing to the local government may not be feasible or acceptable. To put this in context, the city of Depok will need to reallocate USD 2,989,927 or roughly 23-fold of their health budget in 2018 to finance Xpert MTB/RIF expansion over the next four years.

The substantial increase in patient costs related to the use of Xpert MTB/RIF testing must also be considered. Typically, patient costs become a barrier that hinders the utilization of Xpert MTB/RIF testing in developing countries [5, 20], including Indonesia. Although patients enjoy free access to anti-TB drugs, they frequently incur high costs on travel and food and suffer from income losses [3, 35]. Similar studies in other countries have revealed that besides the direct cost of Xpert MTB/RIF testing, transportation remains an issue [29, 35]. In the West Java context, this should be considered as a component that requires subsidies from the government.

Given that the WHO stated that no patient should face catastrophic costs because of TB by 2020 as part of the End TB strategy [4], the study suggests that the formulation of a policy instrument is necessary to support patients with TB regarding high levels of household spending on TB diagnosis and treatment [19, 36]. Additionally, innovative efforts must be enhanced, such as public–private partnerships [9], which have been initiated in West Java. In this regard, engaging the private sector to refer TB suspects to services could lead to detecting other additional positive cases [37] and improving prevention services for latent TB infections [38–40]. Moreover, policy efforts should be in place to ensure demand creation from the patient perspective. Access to Xpert MTB/RIF diagnostic could be increased by involving private health care to provide the test.

Although the central government provides a subsidy of USD 54 per MDR-TB patient to support transportation for getting treatment, it is unclear how the subsidy will improve overall compliance [41]. Furthermore, the lack of follow-ups or no valid data on the number of successful treatments remains a major challenge. Given the resource constraints in a high-burden setting such as West Java, the NTP must create a mechanism on the number of Xpert MTB/RIF machines to be installed, and how to incorporate it into the current diagnostic algorithm. In this regard, the resources would be more efficiently utilized if a systematic plan based on risk factors is developed using Xpert MTB/RIF testing, i.e., patients with previous TB treatment or contact with MDR-TB patients [42]. Meanwhile, since TB patients may live in areas where laboratory infrastructure is insufficient, the DHO must consider improving the network for patient access [33].

Since improving access to TB tests can lead to TB treatment, readiness on the supply side is required as well as an increase in patient compliance. However, the data revealed that the compliance and success rates in West Java are still below the target. Specifically, compliance to treatment decreased from 88.4% in 2016 to 73.9% in 2018, while the success rate of treatment for TB in 2016 remained low at 43% [27].

Finally, local commitment is essential for supporting operational costs, particularly for active case detection, active surveillance, information systems, and programs for ensuring compliance to TB treatment. In this regard, a systematic process of advocacy with the government at all levels is critical. However, unless all of the goals and strategic plans of the central government as well as the action plans at the sub-national level are ensured through political commitments, it is unrealistic for West Java to achieve TB elimination by the year 2030.

There are several limitations of this study that should be noted. First, the analysis of the data obtained from electronic TB registers at the local level might have produced underestimated information, given that the records were not fully maintained as expected. The target of presumptive cases was given by West Java PHO such that no epidemiological parameters based on primary data collection to support analysis, test repeat rates, and diagnostic accuracy may influence the result for budget impact. Second, the sub-national data was obtained from three districts, whereas the patient-level data was obtained from one region, even though the variation in West Java is wide. Ideally, the stratified random sampling approach is selected within the following strata: level of care, ownership (public or private), and diagnostic intervention available [43]. The current study captured data for health system costs from health centers and public hospitals only, while those for patient costs were collected from health centers and private hospitals. For a more detailed analysis, the sample size should be bigger. Third, this study did not cover HIV, pediatric, and TB MDR cases. Fourth, fiscal capacity in all districts in West Java is relatively similar; however, district readiness may be varied. Moreover, we were unable to obtain information on how local authorities consider the readiness of facilities. Finally, unless we perform a systematic tracking of TB funds in certain regions, the fragmented health budgeting system in Indonesia will continue to pose a challenge. Meanwhile, it will provide an opportunity for lessons to be learned in terms of understanding the commitment of the local government.

## Conclusion

This study has added to the current limited knowledge on how Indonesia could develop a plan to increase TB case detection at the sub-national level. In order to accelerate the achievement of the case detection target up to 2024, West Java needs additional Xpert MTB/RIF machines and significant funds. Scaling up the TB tests also requires a commitment, based on careful consideration from both provider and patient perspectives. Moreover, support from the central government is necessary, with careful prioritization, since even one case detection can be quite costly. Finally, to gain support from the sub-national authorities, the NTP requires support from the Ministry of Internal Affairs, who can guide the local authorities to strongly support the MOH decree on TB diagnosis.

## Supporting information

S1 Appendix Informed consent and questionnaire. (PDF)

S1 Table TB cases and fiscal index profile in West Java. (PDF)

S2 Table Estimated cases, case detection targets, and TB presumptive cases in West Java, 2019–2024. (PDF)

S1 Fig Graph of uncertainty analysis. (PDF)
