# Supplementary material for: Cost and affordability of scaling up tuberculosis diagnosis using Xpert MTB/RIF testing in West Java, Indonesia
Source: PLoS One. 2022 Mar 10;17(3):e0264912. doi: 10.1371/journal.pone.0264912 (PMC8912192; doi:10.1371/journal.pone.0264912)
Supplement: S1 Appendix — (PDF) [file pone.0264912.s001.pdf]

S1 Appendix. Informed Consent and Questionnaire

Informed Consent (English) **Patient code number** | | |

INFORMATION OF THE STUDY AND INFORMED CONCENT

Research Title : **Cost Analysis of TB Diagnosis**
Researcher : Dr. drg. Mardiati Nadjib, M.Sc

Dear respondent (sir/ madam),

I am a researcher from Faculty of Public Health, Universitas Indonesia, conducting a study on cost of TB diagnosis in public health center (Puskesmas) and/or hospital. The benefit of this research is to provide inputs related to cost and updated TB diagnosis algorithm compared to previous algorithm and determine whether this TB diagnosis test is effective/ less costly
The research focuses on data of patients' spending (in Rupiah) collected through an interview. The interview will be recorded to help researcher keep all information, and interviewer will make a note on your answer/ reponse. All collected data from this research is confidential and anonimous, no name will appear in our study result. The interview will take approximately 15-30 minutes. You may agree or disagree to participate, and you are welcome to ask more information about this research.
Thank you for your participation.

On behalf of the PI (Dr. drg. Mardiati Nadjib MSC),

Interviewer name and signature: ....

Date:

RESPONDENT CONSENT

After listening description about this study and I understand about the objectives of this research, I am aware that I comply to commit myself in this research as respondent. If more information is needed , I agree to provide more info.

Name :____

Address :____

HP Number :____

Signature :____

Date:

Informed Consent (Bahasa) **kode pasien**

LEMBAR INFORMASI UNTUK CALON RESPONDEN

Judul Penelitian **: Cost Analysis of TB Diagnosis**
Peneliti : Dr. drg. Mardiati Nadjib, M.Sc

Bapak/Ibu yang terhormat,

Saya peneliti dari Fakultas Kesehatan Masyarakat Universitas Indonesia, saat ini sedang melakukan penelitian tentang pembiayaan yang dikeluarkan pasien selama melakukan pemeriksaan/ tes diagnosa penyakit Tuberkulosis di Puskesmas, dan/atau rumah sakit. Manfaat penelitian adalah memberikan masukan terkait biaya dan hasil dari algoritma diagnosis TB saat ini dibandingkan dengan algoritma sebelumnya untuk menemukan algoritma yang paling efektif dan terjangkau.
Penelitian ini akan berfokus pada pengeluaran (dalam satuan rupiah) melalui teknik wawancara. Wawancara akan direkam dengan *recorder* untuk memudahkan kami menyimpan seluruh informasi yang diperoleh, serta akan dilakukan pencatatan atas jawaban responden.
Semua data pada penelitian ini akan dirahasiakan sehingga tidak memungkinkan orang lain menghubungkannya dengan Anda. Semua informasi yang diberikan hanya akan digunakan untuk kepentingan penelitian, tidak akan mempengaruhi pelayanan Puskesmas dan rumah sakit terhadap informan penelitian. Total waktu yang diperlukan sekitar 15-30 menit. Anda bebas memutuskan apakah bersedia berpartisipasi dalam penelitian ini atau tidak. Anda diberi kesempatan untuk menanyakan semua hal yang belum jelas mengenai penelitian ini.
Terima kasih atas perhatian dan kerja sama yang baik dari Bapak/Ibu.

Peneliti, Dr. drg. Mardiati Nadjib MSC

Pewawancara: ....

Tanggal:

LEMBAR PERSETUJUAN

Setelah mendapat penjelasan tentang maksud dan tujuan serta memahami penelitian di atas, dengan ini saya menyatakan kesediaan menjadi responden dengan memberikan jawaban yang sebenar-benarnya dan apabila ada kekurangan di kemudian hari, maka saya bersedia dihubungi untuk dimintai informasi lebih lanjut.

Nama :____

Alamat :____

Nomor HP :____

Tanda Tangan :____

Tanggal:

Research Team Faculty of Public Health – Universitas Indonesia, Kampus UI Depok; Phone/Fax. +62 21 7875576; Email:mardiatinadjib@gmail.com

DIRECT NON-MEDICAL AND INDIRECT COST QUESTIONNAIRE

PART I. Demographic dan Clinical Data

A. IDENTIFICATION

1. Research location : Hospital/ Puskesmas ……………………………………….......
2. Identity*
   a. Patient's name : ………………………………………………………………………......
   b. Ownership of ID : 1☐ Yes 0☐ No
   c. Residency based on ID : 1☐ Depok 0☐ Non-Depok
   d. Address : ………………………………………………………………………......
   e. Contact number : ☐☐☐☐☐☐☐☐☐☐☐☐☐

*Patient's identity is a required information for identification in this research and it is confidential. In the analysis and reporting, patient is only identified by ID number or medical record number.

Information about non-Depok residential and unavailability of BPJS, please include information about how patient will continue TB treatment

Eligibility Criteria (a)	Eligibility Criteria (b)
1. a. Date of Birth: ………/………/……………. (dd/mm/yyyy)<br>b. Age:…… years, ……… months 99☐ No data	Patient is **more than 15 years old**?<br>1☐ Yes 0☐ No
2. Diagnosis – ICD Code ☐.☐☐☐ (Only for hospital)	Patient was diagnosed with pulmonary TB<br>1☐ Yes 0☐ No<br>2☐ Not diagnosed yet
3. Date of diagnosed with TB or expected date of diagnosis result *:<br>………/………/……………. (dd/mm/yyyy) 99☐ No data<br>*for new patient which will be diagnosed, give notes when he/she will have result of examination (tomorrow or days/weeks after). For this patient, will be followed up to capture info on the result and will be aksed about additional cost.	Was diagnosed at least 4 weeks before data collection<br>1☐ Yes 0☐ No<br>2☐ Not diagnosed yet
4. SSM Examination 1☐ Yes 0☐ No 99☐ No data<br>If yes, proceeded in: …………………. 99☐ No data<br>………/………/……………. (dd/mm/yyyy) 99☐ No data	Patient had **TB examination with Microscope** before visiting to the sample health facility?<br>1☐ Yes 0☐ No
5. X-ray examination 1☐ Yes 0☐ No 99☐ No data<br>If yes, proceeded in: …………………. 99☐ No data<br>………/………/……………. (dd/mm/yyyy) 99☐ No data	Patient had **TB examination with X-ray** before visiting to the sample health facility?<br>1☐ Yes 0☐ No

If question number 3 in column (b) is **YES** by non new patients group

Then patient is eligible to include in this research 1☐

Enumerator	Verification Officer	Data Entry
Name : ______________ Signature:<br>Date : ____/_____/2019<br>(dd/mm/yyyy)	Name : ______________ Signature:<br>Date : ____/_____/2019<br>(dd/mm/yyyy)	Name : ______________ Signture:<br>Date : ____/_____/2019<br>(dd/mm/yyyy)

Research Team Faculty of Public Health – Universitas Indonesia, Kampus UI Depok; Phone/Fax. +62 21 7875576; Email:mardiatinadjib@gmail.com

If question number 3 in column (b) is **NO** by non new patients group
Then patient is not eligible to include in this research 2☐

B. DEMOGRAPHIC DATA

1.	Gender	:	1☐ man	2☐ woman		
2.	Education	:	1☐ No School	3☐ Secondary	5☐ D1/D2/D3	99☐ No data
			2☐ Primary	4☐ High School	6☐ S1/S2/S3	
3.	Occupation	:	1☐ Civil Servants	4☐ Housewife		99☐ No data
			2☐ Self-Employed	5☐ Unemployed		7 ☐ Student
			3☐ Employee	6☐ Other, (Please specify) ...………………………..		
4.	Smoking	:	1☐ Yes (actively)	2☐ Yes (has stopped)	3☐Never smoking	
			99☐ No data			
5.	Body Mass Index	:	……………………. kg/m²			
6.	Having Loss Weight	:	1☐ Yes 0☐ No	If yes, by how much? ……… kg		
7.	Having any families/relatives diagnosed with TB	:	1☐ Yes (cured)	2☐ Yes (in treatment)	3☐No	
			99☐ No data	Note: …………………………………		
8.	First time visiting health care facility for TB treatment	:	Referred : 0☐ No	1☐ **Yes**, from……...........……….		99☐ No data
			**if Yes,** By:	1☐ Emergency Unit	2☐ Outpatient	99☐ No data

Enumerator	Verification Officer	Data Entry
Name : ______________ Signature:<br>Date : ____/_____/2019<br>(dd/mm/yyyy)	Name : ______________ Signature:<br>Date : ____/_____/2019<br>(dd/mm/yyyy)	Name : ______________ Signture:<br>Date : ____/_____/2019<br>(dd/mm/yyyy)

PART II. Cost

A. PATIENT'S HEALTH INSURER STATUS

1. Do you have a health insurance/ are you covered by an insurance scheme?

   0☐ No, who is responsible for your health bill payment: ....

   1☐ Yes, what kind of insurance you use:

1☐ BPJS/JKN	4☐ Health Insurance	7☐ Private Insurance: …………….………………
2☐ Jamsostek	5☐ Askeskin/Jamkesmas	8☐ Lain-lain: …………………..………………
3☐ KJS/JAMKESDA	6☐ Company Insurance	9☐ Lain-lain: …………………..………………

2. Other than covered by health insurance, were you charged for other health care service, such as paying for examination, purchasing sputum pot? Rontgen or another medical checkup?

   0☐ No

   1☐ Yes, how much (not from health insurance) did you paid?

   Rp. ☐☐☐.☐☐☐.☐☐☐,☐☐

   which paid for what services? Please mention the cost:

   a. Administration Rp. ☐☐☐.☐☐☐.☐☐☐,☐☐

   b. Doctor's fee Rp. ☐☐☐.☐☐☐.☐☐☐,☐☐

   c. Diagnostic test Rp. ☐☐☐.☐☐☐.☐☐☐,☐☐

   d. Laboratories Rp. ☐☐☐.☐☐☐.☐☐☐,☐☐

   e. Other, (please specify): Rp. ☐☐☐.☐☐☐.☐☐☐,☐☐

   …………………………………………………………….

   …………………………………………………………….

   …………………………………………………………….

   …………………………………………………………….

   …………………………………………………………….

   …………………………………………………………….

Enumerator	Verification Officer	Data Entry
Name : ______________ Signature:<br>Date : ____/_____/2019<br>(dd/mm/yyyy)	Name : ______________ Signature:<br>Date : ____/_____/2019<br>(dd/mm/yyyy)	Name : ______________ Signture:<br>Date : ____/_____/2019<br>(dd/mm/yyyy)

PART III. Non-Medical Direct Cost

A. PATIENT'S TRANPORTATION COST

1. Transportation cost

Please provide info on frequency, mode of transportation and the cost incurred by patient from the initial visit for TB test until diagnosed as having TB. If you cannot decide the cost because you use your own vehicle, mention the distance (in Kilometer) from your house or referral healthcare facilities to the this HC/ hospital.

Transportation Mode	Frequency (times)	Distance (km)	Unit Cost	Total Cost
a. Private/rental car			Rp. ☐.☐☐☐.☐☐☐	Rp. ☐☐☐.☐☐☐.☐☐☐
b. Motorcycle			Rp. ☐.☐☐☐.☐☐☐	Rp. ☐☐☐.☐☐☐.☐☐☐
c. Bus/public transportation			Rp. ☐.☐☐☐.☐☐☐	Rp. ☐☐☐.☐☐☐.☐☐☐
d. Ambulance			Rp. ☐.☐☐☐.☐☐☐	Rp. ☐☐☐.☐☐☐.☐☐☐
e. Air transportation			Rp. ☐.☐☐☐.☐☐☐	Rp. ☐☐☐.☐☐☐.☐☐☐
f. Train			Rp. ☐.☐☐☐.☐☐☐	Rp. ☐☐☐.☐☐☐.☐☐☐
g. Ojek/ motor taxi			Rp. ☐.☐☐☐.☐☐☐	Rp. ☐☐☐.☐☐☐.☐☐☐
h. Taxi			Rp. ☐.☐☐☐.☐☐☐	Rp. ☐☐☐.☐☐☐.☐☐☐
i.			Rp. ☐.☐☐☐.☐☐☐	Rp. ☐☐☐.☐☐☐.☐☐☐
j.			Rp. ☐.☐☐☐.☐☐☐	Rp. ☐☐☐.☐☐☐.☐☐☐

2. Accommodation Cost

Mention number of accommodation cost (if any) during TB test/ examination process.

Accommodation	Frequency	Unit Cost	Total Cost
a.	☐☐☐	Rp. ☐.☐☐☐.☐☐☐	Rp. ☐☐☐.☐☐☐.☐☐☐
b.	☐☐☐	Rp. ☐.☐☐☐.☐☐☐	Rp. ☐☐☐.☐☐☐.☐☐☐

3. Consumption Cost

Mention consumption cost of patient during TB test/ examination process, not included inpatient consumption cost package (if hospitalized).

Item	Frequency	Unit Cost	Total Cost
a.	☐☐☐	Rp. ☐.☐☐☐.☐☐☐	Rp. ☐☐☐.☐☐☐.☐☐☐

Enumerator	Verification Officer	Data Entry
Name : ______________ Signature:	Name : ______________ Signature:	Name : ______________ Signture:
Date : ____/_____/2019 (dd/mm/yyyy)	Date : ____/_____/2019 (dd/mm/yyyy)	Date : ____/_____/2019 (dd/mm/yyyy)

b.	☐☐☐	Rp. ☐.☐☐☐.☐☐☐	Rp. ☐☐☐.☐☐☐.☐☐☐

B. CAREGIVER'S TRANSPORTATION COST

1. Transportation Cost

Provide info on frequency, transportation mode and cost incurred by caregiver from the initial visit for TB test/examination until diagnosed with TB. If you cannot decide the cost because you use your own vehicle, mention the distance (in Kilometer) from your house or referral healthcare facilities to the HC/ hospital.

Transportation Mode	Frequency (times)	Distance (km)	Unit Cost	Total Cost
k. Private/rented car			Rp. ☐.☐☐☐.☐☐☐	Rp. ☐☐☐.☐☐☐.☐☐☐
l. Motorcycle			Rp. ☐.☐☐☐.☐☐☐	Rp. ☐☐☐.☐☐☐.☐☐☐
m. Bus/public transportation			Rp. ☐.☐☐☐.☐☐☐	Rp. ☐☐☐.☐☐☐.☐☐☐
n. Ambulance			Rp. ☐.☐☐☐.☐☐☐	Rp. ☐☐☐.☐☐☐.☐☐☐
o. Air transportation			Rp. ☐.☐☐☐.☐☐☐	Rp. ☐☐☐.☐☐☐.☐☐☐
p. Train			Rp. ☐.☐☐☐.☐☐☐	Rp. ☐☐☐.☐☐☐.☐☐☐
q. Ojek			Rp. ☐.☐☐☐.☐☐☐	Rp. ☐☐☐.☐☐☐.☐☐☐
r. Taxi			Rp. ☐.☐☐☐.☐☐☐	Rp. ☐☐☐.☐☐☐.☐☐☐
s.			Rp. ☐.☐☐☐.☐☐☐	Rp. ☐☐☐.☐☐☐.☐☐☐
t.			Rp. ☐.☐☐☐.☐☐☐	Rp. ☐☐☐.☐☐☐.☐☐☐

2. Accommodation Cost

Mention number of accommodation cost of caregiver (if any) during TB test/ examination process.

Accommodation	Frequency	Unit Cost	Total Cost
c.	☐☐☐	Rp. ☐.☐☐☐.☐☐☐	Rp. ☐☐☐.☐☐☐.☐☐☐
d.	☐☐☐	Rp. ☐.☐☐☐.☐☐☐	Rp. ☐☐☐.☐☐☐.☐☐☐

3. Meals

Mention cost to buy meals for caregiver during TB test/ examination process, not included inpatient cost package (if hospitalized).

Item	Frequency	Unit Cost	Total Cost

Enumerator	Verification Officer	Data Entry
Name : ______________ Signature:	Name : ______________ Signature:	Name : ______________ Signture:
Date : ____/_____/2019 (dd/mm/yyyy)	Date : ____/_____/2019 (dd/mm/yyyy)	Date : ____/_____/2019 (dd/mm/yyyy)

c.	☐☐☐	Rp. ☐.☐☐☐.☐☐☐	Rp. ☐☐☐.☐☐☐.☐☐☐
d.	☐☐☐	Rp. ☐.☐☐☐.☐☐☐	Rp. ☐☐☐.☐☐☐.☐☐☐

C. OTHER PATIENT COST INCURRED

Type	Yes/ no	Unit Cost	Total Cost
a. Laundry	1☐ Yes 2☐ No	Rp. ☐.☐☐☐.☐☐☐	Rp. ☐☐☐.☐☐☐.☐☐☐
b. Drugs/supplements	1☐ Yes 2☐ No	Rp. ☐.☐☐☐.☐☐☐	Rp. ☐☐☐.☐☐☐.☐☐☐
c. Traditional medicine	1☐ Yes 2☐ No	Rp. ☐.☐☐☐.☐☐☐	Rp. ☐☐☐.☐☐☐.☐☐☐
d. Communication	1☐ Yes 2☐ No	Rp. ☐.☐☐☐.☐☐☐	Rp. ☐☐☐.☐☐☐.☐☐☐
e. Sesajen/offering	1☐ Yes 2☐ No	Rp. ☐.☐☐☐.☐☐☐	Rp. ☐☐☐.☐☐☐.☐☐☐
f. Consultation	1☐ Yes 2☐ No	Rp. ☐.☐☐☐.☐☐☐	Rp. ☐☐☐.☐☐☐.☐☐☐
g. Other…...	1☐ Yes 2☐ No	Rp. ☐.☐☐☐.☐☐☐	Rp. ☐☐☐.☐☐☐.☐☐☐
h. ………..	1☐ Yes 2☐ No	Rp. ☐.☐☐☐.☐☐☐	Rp. ☐☐☐.☐☐☐.☐☐☐
i.	1☐ Yes 2☐ No	Rp. ☐.☐☐☐.☐☐☐	Rp. ☐☐☐.☐☐☐.☐☐☐
j.		Rp. ☐.☐☐☐.☐☐☐	Rp. ☐☐☐.☐☐☐.☐☐☐

D. OTHER COST PAID BY/OR CAREGIVER

Type	Status	Unit Cost	Total Cost
k. Laundry	1☐ Yes 2☐ No	Rp. ☐.☐☐☐.☐☐☐	Rp. ☐☐☐.☐☐☐.☐☐☐
l. Drugs/supplements	1☐ Yes 2☐ No	Rp. ☐.☐☐☐.☐☐☐	Rp. ☐☐☐.☐☐☐.☐☐☐
m. Traditional drugs	1☐ Yes 2☐ No	Rp. ☐.☐☐☐.☐☐☐	Rp. ☐☐☐.☐☐☐.☐☐☐
n. Communication	1☐ Yes 2☐ No	Rp. ☐.☐☐☐.☐☐☐	Rp. ☐☐☐.☐☐☐.☐☐☐
o. Sesajen/offering	1☐ Yes 2☐ No	Rp. ☐.☐☐☐.☐☐☐	Rp. ☐☐☐.☐☐☐.☐☐☐
p. Consultation	1☐ Yes 2☐ No	Rp. ☐.☐☐☐.☐☐☐	Rp. ☐☐☐.☐☐☐.☐☐☐
q. Other…...	1☐ Yes 2☐ No	Rp. ☐.☐☐☐.☐☐☐	Rp. ☐☐☐.☐☐☐.☐☐☐
r. ………..	1☐ Yes 2☐ No	Rp. ☐.☐☐☐.☐☐☐	Rp. ☐☐☐.☐☐☐.☐☐☐

Enumerator	Verification Officer	Data Entry
Name : ______________ Signature:<br>Date : ____/_____/2019<br>(dd/mm/yyyy)	Name : ______________ Signature:<br>Date : ____/_____/2019<br>(dd/mm/yyyy)	Name : ______________ Signture:<br>Date : ____/_____/2019<br>(dd/mm/yyyy)

s.	1☐ Yes 2☐ No	Rp. ☐.☐☐☐.☐☐☐	Rp. ☐☐☐.☐☐☐.☐☐☐
t.		Rp. ☐.☐☐☐.☐☐☐	Rp. ☐☐☐.☐☐☐.☐☐☐

**Enumerator**	**Verification Officer**	**Data Entry**
Name : ______________ Signature:<br>Date : ____/_____/2019<br>(dd/mm/yyyy)	Name : ______________ Signature:<br>Date : ____/_____/2019<br>(dd/mm/yyyy)	Name : ______________ Signture:<br>Date : ____/_____/2019<br>(dd/mm/yyyy)

PART IV. Indirect Cost

A. PRODUCTIVITY LOSS (PATIENT)

1. Were there any loss workdays during TB diagnosis process?
   1☐ Yes 0☐ No
2. If Yes, how many days? ☐☐☐ days
3. Occupation and/or activity
   1☐ Full time worker
   2☐ Part time worker
   3☐ Self-employed
   4☐ Unemployed
   5☐ Student
4. Frequency of salary received?
   1☐ Daily 2☐ Weekly 3☐ Monthly
5. How much on average your take home income per month?
   Rp. ☐☐☐.☐☐☐.☐☐☐ per month, or:
   1☐ < Rp. 1.000.000 per month
   2☐ Rp. 1.000.000 – Rp. 3.000.000 per month
   3☐ Rp. 3.000.000 – Rp. 5.000.000 per month
   4☐ Rp. 5.000.000 – Rp. 10.000.000 per month
   5☐ > Rp. 10.000.000 per month
6. If the income is not on regular basis, how much on average per month?
   1☐ daily Rp. ☐☐☐.☐☐☐.☐☐☐
   2☐ weekly Rp. ☐☐☐.☐☐☐.☐☐☐
7. Is there any additional income, how much?
   Rp. ☐☐☐.☐☐☐.☐☐☐ per day, or Rp. ☐☐☐.☐☐☐.☐☐☐ per month
8. How many visits were required / done for your TB tset/ examination?
   1☐ one-time 3☐ three times 5☐ more, …………
   2☐ two times 4☐ four times
9. If more than once visit of test/ examination, when are you scheduled to visit again?
   1☐ a day after 1☐ three days after 1☐ more, …………
   1☐ a day after tomorrow 1☐ a week after
10. How many days was spent for diagnostic testing? ☐☐☐ days
11. During TB examination/TB diagnosis (before treatment), how much cost was lost? (because of loss workdays)
    Rp. ☐☐☐.☐☐☐.☐☐☐

Enumerator	Verification Officer	Data Entry
Name : ______________ Signature:<br>Date : ____/_____/2019<br>(dd/mm/yyyy)	Name : ______________ Signature:<br>Date : ____/_____/2019<br>(dd/mm/yyyy)	Name : ______________ Signture:<br>Date : ____/_____/2019<br>(dd/mm/yyyy)

B. PRODUCTIVITY LOSS (CAREGIVER)

1. Were there any loss workdays of caregiver during TB examination or TB diagnosis process?
   1☐ Yes 0☐ No
2. If Yes, how many days? ☐☐☐ days
3. Occupation and/or activity
   1☐ Full time worker
   2☐ Part time worker
   3☐ Self-employed
   4☐ Unemployed
   5☐ Student
4. Frequency of salary received?
   1☐ Daily 2☐ Weekly 3☐ Monthly
5. How much on average take home income per month?
   Rp. ☐☐☐.☐☐☐.☐☐☐ per month, or:
   1☐ < Rp. 1.000.000 per month
   2☐ Rp. 1.000.000 – Rp. 3.000.000 per month
   3☐ Rp. 3.000.000 – Rp. 5.000.000 per month
   4☐ Rp. 5.000.000 – Rp. 10.000.000 per month
   5☐ > Rp. 10.000.000 per month
6. If the income is not on regular basis, how much on average per month?
   1☐ daily Rp. ☐☐☐.☐☐☐.☐☐☐
   2☐ weekly Rp. ☐☐☐.☐☐☐.☐☐☐
7. Is there any additional income, how much?
   Rp. ☐☐☐.☐☐☐.☐☐☐ per day, or Rp. ☐☐☐.☐☐☐.☐☐☐ per month
8. How many days of caregiver was spent for accompanying patient during TB test/ examination?
   ☐☐☐ days
9. During TB examination/diagnosis process (before treatment), how much cost was lost during accompanying patient? (because of loss of workdays)
   Rp. ☐☐☐.☐☐☐.☐☐☐

THANK YOU VERY MUCH

Enumerator	Verification Officer	Data Entry
Name : _____________ Signature:<br>Date : ____/_____/2019<br>(dd/mm/yyyy)	Name : _____________ Signature:<br>Date : ____/_____/2019<br>(dd/mm/yyyy)	Name : _____________ Signture:<br>Date : ____/_____/2019<br>(dd/mm/yyyy)

Tim Studi Fakultas Kesehatan Masyarakat - Kampus UI Depok; Tel./Fax. +62 21 7875576; Email: info@cheps.or.id

INSTRUMEN BIAYA LANGSUNG NON-MEDIS DAN BIAYA TIDAK LANGSUNG

BAGIAN I. Demografi dan Data Klinis

A. IDENTIFIKASI

1. Lokasi studi : Rumah Sakit/ Puskesmas ……………………………………………......
2. Identitas pasien*
   a. Nama pasien : ……………………………………………………………………………......
   b. Punya KTP : 1☐ Ya 0☐ Tidak
   c. Domisili KTP : 1☐ Depok 0☐ Non-Depok
   d. Alamat tinggal : ……………………………………………………………………………......
   e. Nomor kontak : ☐☐☐☐☐☐☐☐☐☐☐☐☐

*Identitas pasien merupakan kebutuhan untuk kelengkapan informasi pada studi ini dan bersifat rahasia. Dalam analisis data dan pelaporan, pasien hanya teridentifikasi melalui nomor ID pasien atau nomor rekam medis.

Informasi mengenai KTP non Depok dan tidakpunya BPJS mohon ditambahkan bagaimana pasien akan meneruskan pengobatan TB

**Kriteria Eligibilitas (a)**	**Kriteria Eligibilitas (b)**
1. a. Tanggal lahir: ………/………/……………. (dd/mm/yyyy)<br>b. Usia:……… tahun, ……… bulan 99☐ Tidak ada data	Pasien **berusia di atas 18 tahun?**<br>1☐ Ya 0☐ Tidak
2. Diagnosis – Kode ICD ☐.☐☐☐ (Hanya untuk di RS)	Pasien di diagnosis TB Paru<br>1☐ Ya 0☐ Tidak<br>2☐ Belum ada diagnosis
3. Tanggal diagnosis TB atau akan mendapatkan hasil diagnosis*:<br>………/………/……………. (dd/mm/yyyy) 99☐ Tidak ada data<br>*untuk pasien baru yang datang untuk penegakan diagnosa. Beri catatan kapan akan mengambil hasil (keesokan harinya atau beberapa hari/ minggu sesudahnya). Untuk pasien seperti ini diwawancara kembali keesokan harinya saat mengambil hasil, untuk memperoleh informasi biaya tambahan	Diagnosis ditegakkan maksimal 4 minggu sebelum waktu pengumpulan data<br>1☐ Ya 0☐ Tidak<br>2☐ Belum ada diagnosis
4. Pemeriksaan SSM 1☐ Ya 0☐ Tidak 99☐ Tidak ada data<br>Jika Ya, dilakukan di: …………………. 99☐ Tidak ada data<br>………/………/……………. (dd/mm/yyyy) 99☐ Tidak ada data	Pasien **menjalani pemeriksaan TB dengan Mikroskop** sebelum berkunjung ke faskes sampel?<br>1☐ Ya 0☐ Tidak
5. Pemeriksaan Xray 1☐ Ya 0☐ Tidak 99☐ Tidak ada data<br>Jika Ya, dilakukan di: …………………. 99☐ Tidak ada data<br>………/………/……………. (dd/mm/yyyy) 99☐ Tidak ada data	Pasien **menjalani pemeriksaan TB dengan Xray** sebelum berkunjung ke faskes sampel?<br>1☐ Ya 0☐ Tidak

Jika pertanyaan kriteria eligibilitas nomor 3 pada kolom (b) dijawab **YA** pada kelompok bukan pasien baru
Maka pasien eligible untuk dilibatkan dalam studi 1☐
Jika pertanyaan nomor **3** kriteria eligibilitas pada kolom (b) dijawab **Tidak** pada kelompok bukan pasien baru
Maka pasien tidak eligible untuk dilibatkan dalam studi 2☐

**Enumerator**	**Verifikator**	**Data Entry**
Nama : ______________ Paraf:<br>Tanggal : ____/_____/2019<br>(dd/mm/yyyy)	Nama : ______________ Paraf:<br>Tanggal : ____/_____/2017<br>(dd/mm/yyyy)	Nama : ______________ Paraf:<br>Tanggal : ____/_____/2017<br>(dd/mm/yyyy)

B. DEMOGRAFI

1.	Jenis kelamin	:	1☐ Pria	2☐ Wanita	
2.	Pendidikan terakhir	:	1☐ Tidak sekolah	3☐ SMP	5☐ D1/D2/D3 99☐ Tidak ada data
			2☐ SD	4☐ SMA	6☐ S1/S2/S3
3.	Pekerjaan	:	1☐ PNS	4☐ Ibu rumah tangga	99☐ Tidak ada data
			2☐ Wiraswasta	5☐ Tidak bekerja	7 ☐ Pelajar
			3☐ Karyawan	6☐ Lainnya, sebutkan……………………………..	
4.	Status Merokok	:	1☐ Ya (masih)	2☐ Ya (sudah berhenti)	3☐Tidak Merokok
			99☐ Tidak ada data		
5.	Indeks Masa Tubuh	:	…………………….		
6.	Mengalami penurunan berat badan	:	1☐ Ya 0☐ Tidak	Berapa kg penurunannya? ………	
7.	Ada keluarga / tetangga dengan penyakit TB	:	1☐ Ya (sembuh)	2☐ Ya (masa perawatan)	3☐Tidak ada
			99☐ Tidak ada data	Keterangan : …………………………………	
8.	Pertama kali berkunjung Ke faskes utk TB	:	Rujukan : 0☐ Tidak	1☐ **Ya**, dari……………..	99☐ Tidak ada data
			**jika ya** Melalui: 1☐ UGD	1 ☐ Rawat jalan/polilinik	99☐ Tidak ada data

Enumerator	Verifikator	Data Entry
Nama : _____________ Paraf:	Nama : _____________ Paraf:	Nama : _____________ Paraf:
Tanggal : ____/_____/2019 (dd/mm/yyyy)	Tanggal : ____/_____/2017 (dd/mm/yyyy)	Tanggal : ____/_____/2017 (dd/mm/yyyy)

BAGIAN II. Pembiayaan

A. STATUS PENJAMIN/PENANGGUNG BIAYA PASIEN

1. Apakah anda (pasien) peserta asuransi atau jaminan kesehatan?

   0☐ Tidak, siapa yang menanggung biaya pelayanan kesehatan: ....

   1☐ Ya, mohon sebutkan jenis jaminan yang digunakan untuk perawatan Bapak/Ibu:

1☐ BPJS/JKN	4☐ Askes	7☐ Asuransi swasta:…………………..………………
2☐ Jamsostek	5☐ Askeskin/Jamkesmas	8☐ Lain-lain:…………………..………………
3☐ KJS/JAMKESDA	6☐ Jaminan perusahaan	9☐ Lain-lain:…………………..………………

2. Selain biaya yang telah ditanggung asuransi, apakah Anda juga mengeluarkan biaya pelayanan kesehatan lain, misalnya harus membayar pemeriksaan, membeli pot sputum? Rontgen atau periksa tes lain?

   0☐ Tidak

   1☐ Ya, berapa biaya (di luar tanggungan asuransi) yang harus Anda keluarkan?

   Rp. ☐☐☐.☐☐☐.☐☐☐,☐☐

   yang digunakan untuk membayar apa saja? Mohon disebutkan jumlahnya:

   a. Administrasi Rp. ☐☐☐.☐☐☐.☐☐☐,☐☐

   b. Biaya dokter Rp. ☐☐☐.☐☐☐.☐☐☐,☐☐

   c. Tes diagnostik Rp. ☐☐☐.☐☐☐.☐☐☐,☐☐

   d. Laboratorium Rp. ☐☐☐.☐☐☐.☐☐☐,☐☐

   e. Lainnya, meliputi: Rp. ☐☐☐.☐☐☐.☐☐☐,☐☐

   ……………………………………………………………….
   ……………………………………………………………….
   ……………………………………………………………….
   ……………………………………………………………….
   ……………………………………………………………….
   ……………………………………………………………….

Enumerator	Verifikator	Data Entry
Nama : ______________ Paraf:<br>Tanggal : ____/_____/2019<br>(dd/mm/yyyy)	Nama : ______________ Paraf:<br>Tanggal : ____/_____/2017<br>(dd/mm/yyyy)	Nama : ______________ Paraf:<br>Tanggal : ____/_____/2017<br>(dd/mm/yyyy)

BAGIAN III. Biaya Langsung Non-Medis

A. BIAYA TRANSPORTASI PASIEN

1. Biaya Transportasi

Sebutkan jumlah frekuensi dan besaran biaya transportasi yang dikeluarkan oleh/untuk pasien dibatasi dari pertama kali kunjungan untuk pemeriksaan TB hingga diagnosis ditegakkan. Apabila anda tidak dapat menentukan biaya satuan karena menggunakan alat transportasi pribadi, sebutkan jarak tempuh (dalam Kilometer) dari rumah atau faskes perujuk hingga ke Rumah Sakit.

Jenis Moda	Frekuensi (kali)	Jarak (km)	Biaya Satuan	Total Biaya
a. Mobil pribadi/sewa			Rp. ☐.☐☐☐.☐☐☐	Rp. ☐☐☐.☐☐☐.☐☐☐
b. Motor			Rp. ☐.☐☐☐.☐☐☐	Rp. ☐☐☐.☐☐☐.☐☐☐
c. Bus/angkutan umum			Rp. ☐.☐☐☐.☐☐☐	Rp. ☐☐☐.☐☐☐.☐☐☐
d. Ambulan			Rp. ☐.☐☐☐.☐☐☐	Rp. ☐☐☐.☐☐☐.☐☐☐
e. Transportasi udara			Rp. ☐.☐☐☐.☐☐☐	Rp. ☐☐☐.☐☐☐.☐☐☐
f. Kereta			Rp. ☐.☐☐☐.☐☐☐	Rp. ☐☐☐.☐☐☐.☐☐☐
g. Ojek			Rp. ☐.☐☐☐.☐☐☐	Rp. ☐☐☐.☐☐☐.☐☐☐
h. Taksi			Rp. ☐.☐☐☐.☐☐☐	Rp. ☐☐☐.☐☐☐.☐☐☐
i. Parkir kendaraan			Rp. ☐.☐☐☐.☐☐☐	Rp. ☐☐☐.☐☐☐.☐☐☐
j.			Rp. ☐.☐☐☐.☐☐☐	Rp. ☐☐☐.☐☐☐.☐☐☐

2. Biaya Akomodasi

Sebutkan jumlah dan besaran pengeluaran untuk akomodasi pasien (jika ada) selama proses pemeriksaan TB.

Jenis akomodasi	Frekuensi	Biaya Satuan	Total Biaya
a.	☐☐☐	Rp. ☐.☐☐☐.☐☐☐	Rp. ☐☐☐.☐☐☐.☐☐☐
b.	☐☐☐	Rp. ☐.☐☐☐.☐☐☐	Rp. ☐☐☐.☐☐☐.☐☐☐

3. Biaya Makan

Sebutkan biaya makan yang dikeluarkan pasien selama proses pemeriksaan TB, diluar paket yang sudah termasuk dalam rawat inap (jika di rawat).

Item	Frekuensi	Biaya Satuan	Total Biaya
a.	☐☐☐	Rp. ☐.☐☐☐.☐☐☐	Rp. ☐☐☐.☐☐☐.☐☐☐
b.	☐☐☐	Rp. ☐.☐☐☐.☐☐☐	Rp. ☐☐☐.☐☐☐.☐☐☐

Enumerator	Verifikator	Data Entry
Nama : ______________ Paraf:<br>Tanggal : ____/_____/2019<br>(dd/mm/yyyy)	Nama : ______________ Paraf:<br>Tanggal : ____/_____/2017<br>(dd/mm/yyyy)	Nama : ______________ Paraf:<br>Tanggal : ____/_____/2017<br>(dd/mm/yyyy)

B. BIAYA TRANSPORTASI PENDAMPING PASIEN

1. Biaya Transportasi

Sebutkan jumlah frekuensi dan besaran biaya transportasi yang dikeluarkan oleh/untuk pendamping pasien dibatasi dari pertama kali kunjungan untuk pemeriksaan TB hingga diagnosis ditegakkan. Apabila anda tidak dapat menentukan biaya satuan karena menggunakan alat transportasi pribadi, sebutkan jarak tempuh (dalam Kilometer) dari rumah atau faskes perujuk hingga ke Rumah Sakit.

Jenis Moda	Frekuensi (kali)	Jarak (km)	Biaya Satuan	Total Biaya
k. Mobil pribadi/sewa			Rp. ☐.☐☐☐.☐☐☐	Rp. ☐☐☐.☐☐☐.☐☐☐
l. Motor			Rp. ☐.☐☐☐.☐☐☐	Rp. ☐☐☐.☐☐☐.☐☐☐
m. Bus/angkutan umum			Rp. ☐.☐☐☐.☐☐☐	Rp. ☐☐☐.☐☐☐.☐☐☐
n. Ambulan			Rp. ☐.☐☐☐.☐☐☐	Rp. ☐☐☐.☐☐☐.☐☐☐
o. Transportasi udara			Rp. ☐.☐☐☐.☐☐☐	Rp. ☐☐☐.☐☐☐.☐☐☐
p. Kereta			Rp. ☐.☐☐☐.☐☐☐	Rp. ☐☐☐.☐☐☐.☐☐☐
q. Ojek			Rp. ☐.☐☐☐.☐☐☐	Rp. ☐☐☐.☐☐☐.☐☐☐
r. Taksi			Rp. ☐.☐☐☐.☐☐☐	Rp. ☐☐☐.☐☐☐.☐☐☐
s. Parkir kendaraan			Rp. ☐.☐☐☐.☐☐☐	Rp. ☐☐☐.☐☐☐.☐☐☐
t.			Rp. ☐.☐☐☐.☐☐☐	Rp. ☐☐☐.☐☐☐.☐☐☐

2. Biaya Akomodasi

Sebutkan jumlah dan besaran pengeluaran untuk akomodasi pendamping pasien (jika ada) selama proses pemeriksaan TB.

Jenis akomodasi	Frekuensi	Biaya Satuan	Total Biaya
c.	☐☐☐	Rp. ☐.☐☐☐.☐☐☐	Rp. ☐☐☐.☐☐☐.☐☐☐
d.	☐☐☐	Rp. ☐.☐☐☐.☐☐☐	Rp. ☐☐☐.☐☐☐.☐☐☐

3. Biaya Makan

Sebutkan biaya makan yang dikeluarkan pendamping pasien selama proses pemeriksaan TB, diluar paket yang sudah termasuk dalam rawat inap (jika di rawat).

Item	Frekuensi	Biaya Satuan	Total Biaya
c.	☐☐☐	Rp. ☐.☐☐☐.☐☐☐	Rp. ☐☐☐.☐☐☐.☐☐☐
d.	☐☐☐	Rp. ☐.☐☐☐.☐☐☐	Rp. ☐☐☐.☐☐☐.☐☐☐

Enumerator	Verifikator	Data Entry
Nama : ____________ Paraf:<br>Tanggal : ____/_____/2019<br>(dd/mm/yyyy)	Nama : ____________ Paraf:<br>Tanggal : ____/_____/2017<br>(dd/mm/yyyy)	Nama : ____________ Paraf:<br>Tanggal : ____/_____/2017<br>(dd/mm/yyyy)

C. BIAYA LAINNYA YANG DIKELUARKAN OLEH/UNTUK PASIEN

Jenis Moda	Ststus	Biaya Satuan	Total Biaya
a. Laundry	1☐ Ya 2☐ Tidak	Rp. ☐.☐☐☐.☐☐☐	Rp. ☐☐☐.☐☐☐.☐☐☐
b. Obat/supplemen	1☐ Ya 2☐ Tidak	Rp. ☐.☐☐☐.☐☐☐	Rp. ☐☐☐.☐☐☐.☐☐☐
c. Obat tradisional	1☐ Ya 2☐ Tidak	Rp. ☐.☐☐☐.☐☐☐	Rp. ☐☐☐.☐☐☐.☐☐☐
d. Biaya komunikasi	1☐ Ya 2☐ Tidak	Rp. ☐.☐☐☐.☐☐☐	Rp. ☐☐☐.☐☐☐.☐☐☐
e. Sesajen/beliefs	1☐ Ya 2☐ Tidak	Rp. ☐.☐☐☐.☐☐☐	Rp. ☐☐☐.☐☐☐.☐☐☐
f. Jasa perawat	1☐ Ya 2☐ Tidak	Rp. ☐.☐☐☐.☐☐☐	Rp. ☐☐☐.☐☐☐.☐☐☐
g. Lainnya…..	1☐ Ya 2☐ Tidak	Rp. ☐.☐☐☐.☐☐☐	Rp. ☐☐☐.☐☐☐.☐☐☐
h. ………..	1☐ Ya 2☐ Tidak	Rp. ☐.☐☐☐.☐☐☐	Rp. ☐☐☐.☐☐☐.☐☐☐
i.	1☐ Ya 2☐ Tidak	Rp. ☐.☐☐☐.☐☐☐	Rp. ☐☐☐.☐☐☐.☐☐☐
j.		Rp. ☐.☐☐☐.☐☐☐	Rp. ☐☐☐.☐☐☐.☐☐☐

D. BIAYA LAINNYA YANG DIKELUARKAN OLEH/UNTUK PENDAMPING PASIEN

Jenis Moda	Ststus	Biaya Satuan	Total Biaya
k. Laundry	1☐ Ya 2☐ Tidak	Rp. ☐.☐☐☐.☐☐☐	Rp. ☐☐☐.☐☐☐.☐☐☐
l. Obat/supplemen	1☐ Ya 2☐ Tidak	Rp. ☐.☐☐☐.☐☐☐	Rp. ☐☐☐.☐☐☐.☐☐☐
m. Obat tradisional	1☐ Ya 2☐ Tidak	Rp. ☐.☐☐☐.☐☐☐	Rp. ☐☐☐.☐☐☐.☐☐☐
n. Biaya komunikasi	1☐ Ya 2☐ Tidak	Rp. ☐.☐☐☐.☐☐☐	Rp. ☐☐☐.☐☐☐.☐☐☐
o. Sesajen/beliefs	1☐ Ya 2☐ Tidak	Rp. ☐.☐☐☐.☐☐☐	Rp. ☐☐☐.☐☐☐.☐☐☐
p. Jasa perawat	1☐ Ya 2☐ Tidak	Rp. ☐.☐☐☐.☐☐☐	Rp. ☐☐☐.☐☐☐.☐☐☐
q. Lainnya…..	1☐ Ya 2☐ Tidak	Rp. ☐.☐☐☐.☐☐☐	Rp. ☐☐☐.☐☐☐.☐☐☐
r. ………..	1☐ Ya 2☐ Tidak	Rp. ☐.☐☐☐.☐☐☐	Rp. ☐☐☐.☐☐☐.☐☐☐
s.	1☐ Ya 2☐ Tidak	Rp. ☐.☐☐☐.☐☐☐	Rp. ☐☐☐.☐☐☐.☐☐☐
t.		Rp. ☐.☐☐☐.☐☐☐	Rp. ☐☐☐.☐☐☐.☐☐☐

Enumerator	Verifikator	Data Entry
Nama : ______________ Paraf:<br>Tanggal : ____/_____/2019<br>(dd/mm/yyyy)	Nama : ______________ Paraf:<br>Tanggal : ____/_____/2017<br>(dd/mm/yyyy)	Nama : ______________ Paraf:<br>Tanggal : ____/_____/2017<br>(dd/mm/yyyy)

BAGIAN IV. Biaya Tidak Langsung

A. PRODUKTIVITAS YANG HILANG PADA PASIEN SAAT PERIKSA TB

1. Apakah ada hari kerja Anda yang hilang saat selama proses pemeriksaan atau penegakan diagnosis TB?
   1☐ Ya 0☐ Tidak
2. Jika Ya, berapa hari? ☐☐☐ hari
3. Tipe dan aktivitas pekerjaan
   1☐ Pekerjaan tetap/buruh/karyawan
   2☐ Paruh waktu
   3☐ Wiraswasta
   4☐ Tidak bekerja
   5☐ Sekolah/mahasiswa
4. Sistem pembayaran upah/gaji?
   1☐ Harian 2☐Mingguan 3☐Bulanan
5. Berapa perkiraan penghasilan rata-rata dalam satu bulan (*take home income*)?
   Rp. ☐☐☐.☐☐☐.☐☐☐ per bulan, atau:
   1☐ < Rp. 1.000.000 per bulan
   2☐ Rp. 1.000.000 – Rp. 3.000.000 per bulan
   3☐ Rp. 3.000.000 – Rp. 5.000.000 per bulan
   4☐ Rp. 5.000.000 – Rp. 10.000.000 per bulan
   5☐ > Rp. 10.000.000 per bulan
6. Jika pendapatan tidak menentu, berapa rata-rata penghasilan
   1☐ harian Rp. ☐☐☐.☐☐☐.☐☐☐
   2☐ mingguan Rp. ☐☐☐.☐☐☐.☐☐☐
7. Apakah ada tambahan penghasilan lain yang diperoleh, dan berapa jumlahnya?
   Rp. ☐☐☐.☐☐☐.☐☐☐ per hari, atau Rp. ☐☐☐.☐☐☐.☐☐☐ per bulan
8. Berapa kali kunjungan yang dibutuhkan dalam proses pemeriksaan TB
   1☐ satu kali 3☐tiga kali 5☐ lebih…………
   2☐ dua kali 4☐empat kali
9. Jika kunjungan lebih dari satu kali untuk pemeriksaan, kapan pasien dijadwalkan untuk berkunjung kembali
   1☐ keeseokan hari 1☐tiga hari kemudian 1☐ lebih…………
   1☐ dua hari kemudian 1☐satu minggu kemudian
10. Berapa lama total waktu yang dihabiskan selama melakukan tes diagnostik? ☐☐☐ hari
11. Selama proses pemeriksaan/penegakan diagnosis TB (sebelum pengobatan), berapa kira-kira biaya yang hilang? (karena meninggalkan pekerjaan)
    Rp. ☐☐☐.☐☐☐.☐☐☐

Enumerator	Verifikator	Data Entry
Nama : ______________ Paraf:<br>Tanggal : ____/_____/2019<br>(dd/mm/yyyy)	Nama : ______________ Paraf:<br>Tanggal : ____/_____/2017<br>(dd/mm/yyyy)	Nama : ______________ Paraf:<br>Tanggal : ____/_____/2017<br>(dd/mm/yyyy)

B. PRODUKTIVITAS YANG HILANG PADA PENDAMPING PASIEN SAAT PROSES PEMERIKSAAN TB

1. Apakah ada hari kerja Anda yang hilang saat mendampingi pasien dalam proses pemeriksaan/penegakan diagnosis TB?
   1☐ Ya 0☐ Tidak
2. Jika Ya, berapa hari? ☐☐☐ hari
3. Tipe dan aktivitas pekerjaan
   1☐ Pekerjaan tetap/buruh/karyawan
   2☐ Paruh waktu
   3☐ Wiraswasta
   4☐ Tidak bekerja
   5☐ Sekolah/mahasiswa
4. Sistem pembayaran upah/gaji?
   1☐ Harian 2☐Mingguan 3☐Bulanan
5. Berapa perkiraan penghasilan rata-rata dalam satu bulan (*take home income*)?
   Rp. ☐☐☐.☐☐☐.☐☐☐ per bulan, atau:
   1☐ < Rp. 1.000.000 per bulan
   2☐ Rp. 1.000.000 – Rp. 3.000.000 per bulan
   3☐ Rp. 3.000.000 – Rp. 5.000.000 per bulan
   4☐ Rp. 5.000.000 – Rp. 10.000.000 per bulan
   5☐ > Rp. 10.000.000 per bulan
6. Jika pendapatan tidak menentu, berapa rata-rata penghasilan
   1☐ harian Rp. ☐☐☐.☐☐☐.☐☐☐
   2☐ mingguan Rp. ☐☐☐.☐☐☐.☐☐☐
7. Apakah ada tambahan penghasilan lain yang diperoleh, dan berapa jumlahnya?
   Rp. ☐☐☐.☐☐☐.☐☐☐ per hari, atau Rp. ☐☐☐.☐☐☐.☐☐☐ per bulan
8. Berapa lama total waktu untuk mendampingi pasien selama pemeriksaan/penegakan diagnosis TB?
   ☐☐☐ hari
9. Selama proses pemeriksaan/penegakan diagnosis TB (sebelum pengobatan), berapa kira-kira biaya yang hilang selama mendampingi pasien? (karena meninggalkan pekerjaan)
   Rp. ☐☐☐.☐☐☐.☐☐☐

Enumerator	Verifikator	Data Entry
Nama : ______________ Paraf:<br>Tanggal : ____/_____/2019<br>(dd/mm/yyyy)	Nama : ______________ Paraf:<br>Tanggal : ____/_____/2017<br>(dd/mm/yyyy)	Nama : ______________ Paraf:<br>Tanggal : ____/_____/2017<br>(dd/mm/yyyy)
