# Supplementary material for: Cost and affordability of scaling up tuberculosis diagnosis using Xpert MTB/RIF testing in West Java, Indonesia
Source: PLoS One. 2022 Mar 10;17(3):e0264912. doi: 10.1371/journal.pone.0264912 (PMC8912192; doi:10.1371/journal.pone.0264912)
Supplement: S1 Table — (PDF) [file pone.0264912.s002.pdf]

S1 Table. TB Cases and Fiscal Index Profile in West Java

No	District	2017			Health Center (n)	Fiscal Index 2019	Weighted
		Population	TB All cases	TB BTA (+) All cases			
1	Bekasi City	2,859,630	4,144	1526	39	6.916 (Very High)	1
2	Bogor District	5,715,009	10,405	3831	101	5.563 (Very High)	0.7
3	Bandung City	2,497,938	2,339	1003	75	5.486 (Very High)	1.1
4	Bekasi District	3,500,023	1,756	1050	44	4.409 (Very High)	0.7
5	Bandung District	3,657,601	NA	2174	177	3.716 (Very High)	0.7
6	Karawang District	2,316,489	1,726	1116	116	2.880 (Very High)	0.7
7	Depok City	2,254,513	3,734	1580	37	2.544 (Very High)	1
8	Sukabumi District	2,453,498	3,661	2350	58	2.245 (Very High)	0.6
9	Majalengka District	1,193,725	1,662	1162	32	2.191 (Very High)	0.6
10	Garut District	2,588,839	3,069	1494	67	2.092 (Very High)	0.5
11	Indramayu District	1,709,994	1,889	816	61	2.027 (Very High)	0.6
12	Subang District	1,562,509	906	1187	40	1.986 (Very High)	0.7
13	Cianjur District	2,256,589	3,578	1393	206	1.943 (High)	0.5
14	Bogor City	1,081,009	1,409	979	47	1.811 (High)	0.7
15	Cirebon District	2,159,577	3,923	1486	59	1.592 (High)	0.7
16	Tasikmalaya District	1,747,318	2,193	1073	172	1.266 (High)	0.5
17	Purwakarta District	943,337	1,070	537	55	1.214 (High)	0.7
18	West Bandung District	1,666,510	1,505	710	35	1.209 (High)	0.5
19	Tasikmalaya City	661,404	1,520	516	36	1.157 (High)	0.6
20	Cirebon City	313,325	231	399	22	1.141 (High)	0.8
21	Sumedang District	1,146,435	2,030	726	35	1.103 (High)	0.6
22	Cimahi City	601,099	1,802	540	16	1.086 (Moderate)	0.8
23	Kuningan District	1,068,201	2,110	7169	37	1.056 (Moderate)	0.6
24	Sukabumi City	323,788	667	293	15	1.035 (Moderate)	0.7
25	Ciamis District	1,181,981	1,496	978	37	0.928 (Moderate)	0.6
26	Pangandaran District	395,098	380	294	15	0.719 (Low)	0.6
27	Banjar City	182,388	628	124	10	0.430 (Low)	0.7
	West JavaProvince	48,037,827	59,833	36506	1644	3.171 (Very High)	0.7
