# Supplementary material for: Cost and affordability of scaling up tuberculosis diagnosis using Xpert MTB/RIF testing in West Java, Indonesia
Source: PLoS One. 2022 Mar 10;17(3):e0264912. doi: 10.1371/journal.pone.0264912 (PMC8912192; doi:10.1371/journal.pone.0264912)
Supplement: S2 Table — (PDF) [file pone.0264912.s003.pdf]

S2 Table. Estimated Cases and, Case Finding Target, and TB Presumptive Case in West Java in 2019-2024

No	District	2019			2020			2021			2022			2023			2024		
		Estimated Cases	Case finding Target	TB Presumptive	Estimated Cases	Case finding Target	TB Presumptive	Estimated Cases	Case finding Target	TB Presumptive	Estimated Cases	Case finding Target	TB Presumptive	Estimated Cases	Case finding Target	TB Presumptive	Estimated Cases	Case finding Target	TB Presumptive
1	Bekasi City	8748	7873	42514.2	8758	7882	42562.8	8758	7882	42562.8	8748	8310	44874	8737	8301	44825	8727	8291	44771
2	Bogor District	15056	13551	73175	15074	13567	73262	15074	13567	73262	15056	14304	77242	15039	14287	77150	15021	14270	77058
3	Bandung City	8560	7704	41602	8570	7713	41650	8570	7713	41650	8560	8132	43913	8550	8123	43864	8540	8113	43810
4	Bekasi District	8765	7888	42595	8775	7898	42649	8775	7898	42649	8765	8327	44966	8754	8317	44912	8744	8307	44858
5	Bandung District	10024	9022	48719	10036	9032	48773	10036	9032	48773	10024	9523	51424	10012	9512	51365	10000	9500	51300
6	Karawang District	5715	5143	27772	5722	5149	27805	5722	5149	27805	5715	5429	29317	5708	5423	29284	5701	5416	29246
7	Depok City	6965	6268	33847	6973	6276	33890	6973	6276	33890	6965	6616	35726	6956	6609	35689	6948	6601	35645
8	Sukabumi District	5972	5375	29025	5979	5381	29057	5979	5381	29057	5972	5674	30640	5965	5667	30602	5958	5660	30564
9	Majalengka District	2858	2572	13889	2862	2575	13905	2862	2575	13905	2858	2715	14661	2855	2712	14645	2851	2709	14629
10	Garut District	6495	5845	31563	6502	5852	31601	6502	5852	31601	6495	6170	33318	6487	6163	33280	6479	6155	33237
11	Indramayu District	4127	3714	20056	4132	3719	20083	4132	3719	20083	4127	3920	21168	4122	3916	21146	4117	3911	21119
12	Subang District	3605	3244	17518	3609	3248	17539	3609	3248	17539	3605	3424	18490	3600	3420	18468	3596	3416	18446
13	Cianjur District	5408	4868	26287	5415	4873	26314	5415	4873	26314	5408	5138	27745	5402	5132	27713	5396	5126	27680
14	Bogor City	3573	3216	17366	3577	3220	17388	3577	3220	17388	3573	3394	18328	3569	3390	18306	3565	3386	18284
15	Cirebon District	5765	5188	28015	5772	5195	28053	5772	5195	28053	5765	5477	29576	5758	5470	29538	5751	5464	29506
16	Tasikmalaya District	4090	3681	19877	4095	3686	19904	4095	3686	19904	4090	3886	20984	4085	3881	20957	4081	3877	20936
17	Purwakarta District	2287	2058	11113	2289	2060	11124	2289	2060	11124	2287	2172	11729	2284	2170	11718	2281	2167	11702
18	West Bandung District	4197	3777	20396	4202	3782	20423	4202	3782	20423	4197	2987	16130	4192	3982	21503	4187	3978	21481
19	Tasikmalaya City	2127	1914	10336	2129	1916	10346	2129	1916	10346	2127	2020	10908	2124	2018	10897	2122	2015	10881
20	Cirebon City	1074	967	5222	1075	968	5227	1075	968	5227	1074	1020	5508	1073	1019	5503	1072	1018	5497
21	Sumedang	2756	2481	13397	2760	2484	13414	2760	2484	13414	2756	2619	14143	2753	2616	14126	2750	2612	14105

No	District	2019			2020			2021			2022			2023			2024		
		**Estimated Cases**	**Case finding Target**	**TB Presumptive**	**Estimated Cases**	**Case finding Target**	**TB Presumptive**	**Estimated Cases**	**Case finding Target**	**TB Presumptive**	**Estimated Cases**	**Case finding Target**	**TB Presumptive**	**Estimated Cases**	**Case finding Target**	**TB Presumptive**	**Estimated Cases**	**Case finding Target**	**TB Presumptive**
	District																		
22	Cimahi City	1971	1774	9580	1974	1776	9590	1974	1776	9590	1971	1873	10114	1969	1871	10103	1967	1868	10087
23	Kuningan District	2501	2250	12150	2504	2253	12166	2504	2253	12166	2501	2376	12830	2498	2373	12814	2495	2370	12798
24	Sukabumi City	1057	951	5135	1058	952	5141	1058	952	5141	1057	1004	5422	1055	1003	5416	1054	1001	5405
25	Ciamis District	2743	2469	13333	2746	2472	13349	2746	2472	13349	2743	2606	14072	2740	2603	14056	2737	2600	14040
26	Pangandaran District	909	818	4417	910	819	4423	910	819	4423	909	864	4666	908	863	4660	907	862	4655
27	Banjar City	558	502	2711	559	503	2716	559	503	2716	558	530	2862	557	529	2857	557	529	2857
	West Java Province	127,906	115,113	621,610	128,057	115,251	622,355	128,057	115,251	622,355	127,906	120,510	650,754	127,752	121,370	655,398	127,604	121,222	654,599
