# Supplementary material for: Cost and affordability of scaling up tuberculosis diagnosis using Xpert MTB/RIF testing in West Java, Indonesia
Source: PLoS One. 2022 Mar 10;17(3):e0264912. doi: 10.1371/journal.pone.0264912 (PMC8912192; doi:10.1371/journal.pone.0264912)
Supplement: S1 Fig — (PDF) [file pone.0264912.s004.pdf]

# S1 Figure. Uncertainty Result Graphs

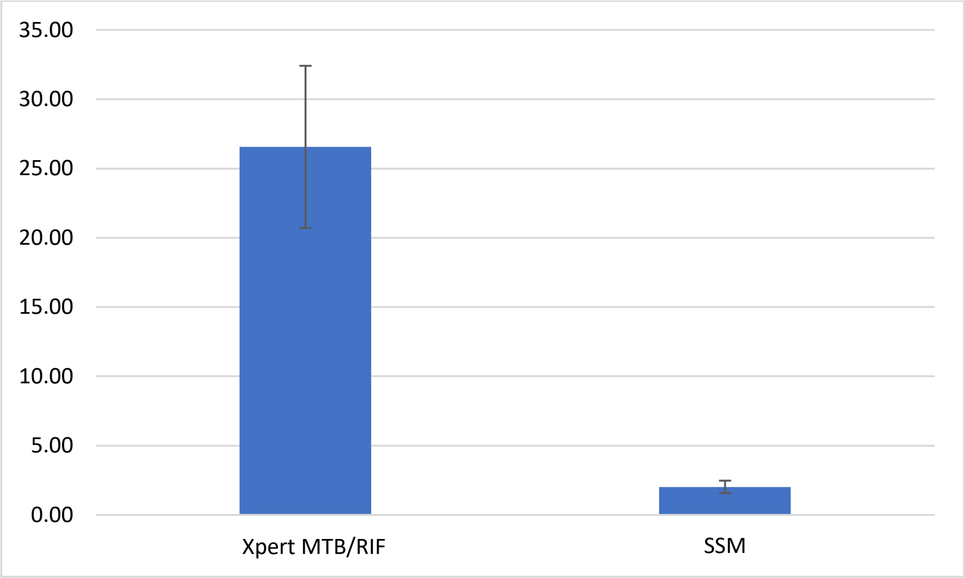


*Graph 1. Adjusted Cost*

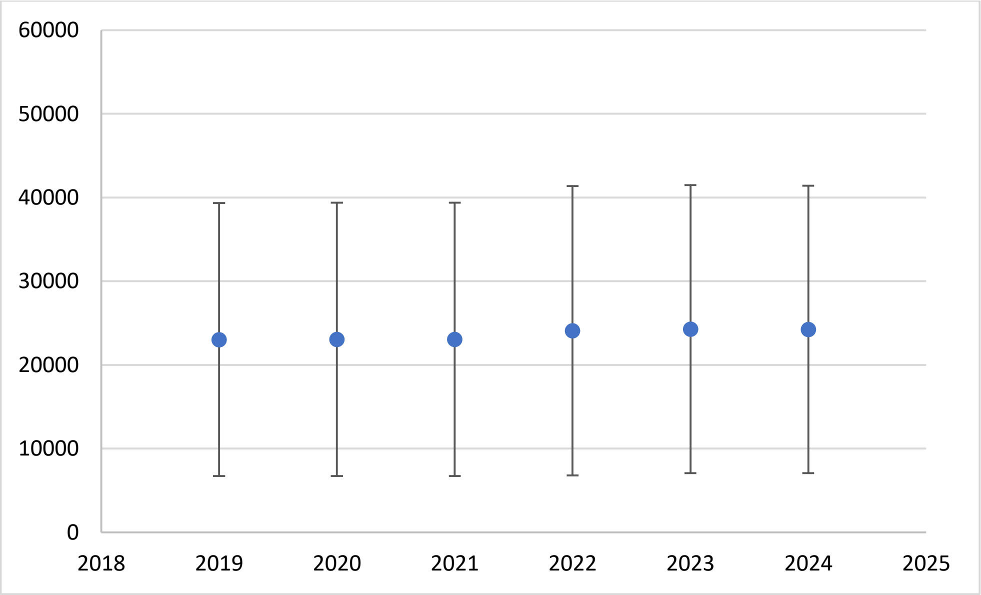


*Graph 2. Average Tb Cases*

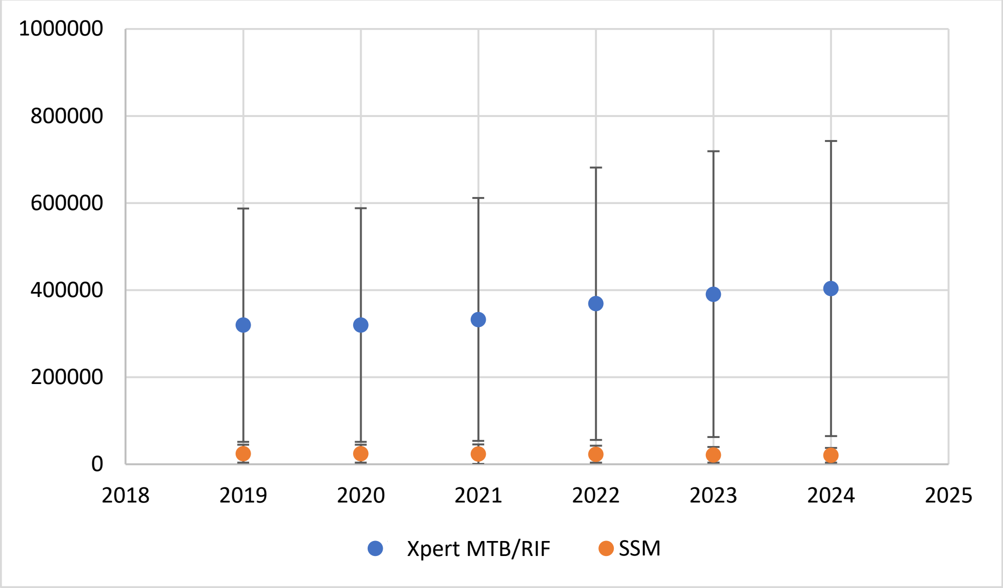


*Graph 3. Total Cost Xpert MTB/RIF (TCM) and SSM Testing*

## Summary Result

*Table 1. Summary Table (by Parameters)*

| Year | Cases (n ± SD) | Total costs (TCM) | Total costs (SSM) |
|---|---|---|---|
| 2019 | 23,022 ± 16,318 | 319,401 ± 24,303 | 24,303 ± 20,406 |
| 2020 | 23,050 ± 16,337 | 319,783 ± 24,332 | 24,332 ± 20,430 |
| 2021 | 23,050 ± 16,337 | 332,575 ± 23,359 | 23,359 ± 22,494 |
| 2022 | 24,102 ± 17,289 | 368,732 ± 22,955 | 22,955 ± 19,613 |
| 2023 | 23,022 ± 16,318 | 319,401 ± 24,303 | 24,303 ± 20,406 |
| 2024 | 23,050 ± 16,337 | 319,783 ± 24,332 | 24,332 ± 20,430 |
